# Evidence of a causal relationship between body mass index and psoriasis: A mendelian randomization study

**DOI:** 10.1371/journal.pmed.1002739

**Published:** 2019-01-31

**Authors:** Ashley Budu-Aggrey, Ben Brumpton, Jess Tyrrell, Sarah Watkins, Ellen H. Modalsli, Carlos Celis-Morales, Lyn D. Ferguson, Gunnhild Åberge Vie, Tom Palmer, Lars G. Fritsche, Mari Løset, Jonas Bille Nielsen, Wei Zhou, Lam C. Tsoi, Andrew R. Wood, Samuel E. Jones, Robin Beaumont, Marit Saunes, Pål Richard Romundstad, Stefan Siebert, Iain B. McInnes, James T. Elder, George Davey Smith, Timothy M. Frayling, Bjørn Olav Åsvold, Sara J. Brown, Naveed Sattar, Lavinia Paternoster

**Affiliations:** 1 Medical Research Council (MRC) Integrative Epidemiology Unit, University of Bristol, Bristol, United Kingdom; 2 Bristol Medical School, Population Health Sciences, University of Bristol, Bristol, United Kingdom; 3 K.G. Jebsen Center for Genetic Epidemiology, Department of Public Health and Nursing, NTNU, Norwegian University of Science and Technology, Trondheim, Norway; 4 Department of Thoracic Medicine, St. Olavs Hospital, Trondheim University Hospital, Trondheim, Norway; 5 Genetics of Complex Traits, Institute of Biomedical and Clinical Science, University of Exeter Medical School, Royal Devon and Exeter Hospital, Exeter, United Kingdom; 6 European Centre for Environment and Human Health, University of Exeter Medical School, The Knowledge Spa, Truro, United Kingdom; 7 Department of Public Health and Nursing, NTNU, Norwegian University of Science and Technology, Trondheim, Norway; 8 Department of Dermatology, St. Olav’s Hospital, Trondheim University Hospital, Trondheim, Norway; 9 Institute of Cardiovascular and Medical Sciences, University of Glasgow, Glasgow, United Kingdom; 10 Department of Mathematics and Statistics, Lancaster University, Lancaster, United Kingdom; 11 Department of Internal Medicine, Division of Cardiology, University of Michigan Medical School, Ann Arbor, Michigan, United States of America; 12 Department of Computational Medicine and Bioinformatics, University of Michigan, Ann Arbor, Michigan, United States of America; 13 Department of Dermatology, University of Michigan, Ann Arbor, Michigan, United States of America; 14 Department of Biostatistics, Center for Statistical Genetics, University of Michigan, Ann Arbor, Michigan, United States of America; 15 Department of Clinical and Molecular Medicine, NTNU, Norwegian University of Science and Technology, Trondheim, Norway; 16 Institute of Infection, Immunity and Inflammation, University of Glasgow, Glasgow, United Kingdom; 17 Ann Arbor Veterans Affairs Hospital, Ann Arbor, Michigan, United States of America; 18 Department of Endocrinology, St. Olav’s Hospital, Trondheim University Hospital, Trondheim, Norway; 19 Skin Research Group, School of Medicine, University of Dundee, Dundee, United Kingdom; 20 Department of Dermatology, Ninewells Hospital and Medical School, Dundee, United Kingdom; King’s College London, UNITED KINGDOM

## Abstract

**Background:**

Psoriasis is a common inflammatory skin disease that has been reported to be associated with obesity. We aimed to investigate a possible causal relationship between body mass index (BMI) and psoriasis.

**Methods and findings:**

Following a review of published epidemiological evidence of the association between obesity and psoriasis, mendelian randomization (MR) was used to test for a causal relationship with BMI. We used a genetic instrument comprising 97 single-nucleotide polymorphisms (SNPs) associated with BMI as a proxy for BMI (expected to be much less confounded than measured BMI). One-sample MR was conducted using individual-level data (396,495 individuals) from the UK Biobank and the Nord-Trøndelag Health Study (HUNT), Norway. Two-sample MR was performed with summary-level data (356,926 individuals) from published BMI and psoriasis genome-wide association studies (GWASs). The one-sample and two-sample MR estimates were meta-analysed using a fixed-effect model. To test for a potential reverse causal effect, MR analysis with genetic instruments comprising variants from recent genome-wide analyses for psoriasis were used to test whether genetic risk for this skin disease has a causal effect on BMI.

Published observational data showed an association of higher BMI with psoriasis. A mean difference in BMI of 1.26 kg/m^2^ (95% CI 1.02–1.51) between psoriasis cases and controls was observed in adults, while a 1.55 kg/m^2^ mean difference (95% CI 1.13–1.98) was observed in children. The observational association was confirmed in UK Biobank and HUNT data sets. Overall, a 1 kg/m^2^ increase in BMI was associated with 4% higher odds of psoriasis (meta-analysis odds ratio [OR] = 1.04; 95% CI 1.03–1.04; *P* = 1.73 × 10^−60^). MR analyses provided evidence that higher BMI causally increases the odds of psoriasis (by 9% per 1 unit increase in BMI; OR = 1.09 (1.06–1.12) per 1 kg/m^2^; *P* = 4.67 × 10^−9^). In contrast, MR estimates gave little support to a possible causal effect of psoriasis genetic risk on BMI (0.004 kg/m^2^ change in BMI per doubling odds of psoriasis (−0.003 to 0.011). Limitations of our study include possible misreporting of psoriasis by patients, as well as potential misdiagnosis by clinicians. In addition, there is also limited ethnic variation in the cohorts studied.

**Conclusions:**

Our study, using genetic variants as instrumental variables for BMI, provides evidence that higher BMI leads to a higher risk of psoriasis. This supports the prioritization of therapies and lifestyle interventions aimed at controlling weight for the prevention or treatment of this common skin disease. Mechanistic studies are required to improve understanding of this relationship.

## Abstract 2

In a mendelian randomization study, Ashley Budu-Aggrey and co-workers study the influence of body mass index on psoriasis.

## Author summary

**Why was this study done?:**

- Psoriasis is a common inflammatory skin disease that has been reported to be associated with obesity. However, the direction of causality has not been established.
- Understanding the causal relationship could inform the management or prevention of disease.

**What did the researchers do and find?:**

- A mendelian randomization (MR) approach was used to investigate the causal relationship between higher body mass index (BMI) and psoriasis.
- Our analysis included data for a total of 753,421 individuals from two of the largest population-based studies available as well as published genome-wide association studies (GWASs).
- We found evidence that higher BMI causally increases the risk of psoriasis, supporting observational reports in previous literature.
- Conversely, there was no evidence to support a causal effect of psoriasis genetic risk upon BMI.

**What do these findings mean?:**

- Our findings suggest that obesity contributes to the pathogenesis of psoriasis, and highlight possible mechanistic relationships.
- If our findings regarding genetically influenced BMI can be extended to elevated BMI that is amenable to modification by diet or behavior, then they could carry health implications.
- Further work will be required to determine the effect of a short-term intervention aimed at reducing BMI upon psoriasis patients after disease onset, ideally within a clinical trial setting.

## Introduction

Psoriasis is a common inflammatory skin disorder that is characterized by erythematous scaly plaques; severe disease is associated with significant impairment in physical and mental health [1]. Psoriasis affects approximately 2% of people within European populations [2], with higher prevalence estimates in northern regions of Europe [3]. The prevalence of disease has also been found to be increasing [4].

Obesity has become one of the leading health issues of the 21st century, with over one-quarter of the United Kingdom population now obese and similarly high obesity levels in many other parts of the world [5]. In addition to clear links of obesity to diabetes and hypertension, observational evidence from epidemiological studies has suggested a relationship between increased weight and psoriasis [6]. Furthermore, a small number of weight loss interventions have been shown to improve psoriasis and increase responsiveness to treatment [7–9]. Hypothetically, obesity could promote skin inflammation, or vice versa [10], but skin disease can also lead to a reduced participation in physical activity, resulting in weight gain. A clearer understanding of the cutaneous and systemic metabolic effects associated with obesity and psoriasis is an essential prerequisite to define treatment and prevention strategies for these prevalent public health issues.

Causality can be investigated with mendelian randomization (MR), which uses genetic variants to, in effect, randomly allocate individuals to groups based on genotype (analogous to a randomized trial) [11]. At conception, genetic variants are expected to be randomly allocated from parents to offspring. Therefore, confounding and reverse causation, common limitations of observational studies, can be avoided by using genetic variants as instrumental variables to estimate the causal effect of a risk factor upon an outcome of interest [11–13]. Genome-wide association studies (GWASs) of body mass index (BMI) in the Genetic Investigation of Anthropometric Traits (GIANT) consortium have identified 97 loci (accounting for 2.7% of the variance of this trait) and made full summary statistics available [14] (a recent study has increased this to 716 loci, explaining 5.0% of the variance [15]). GWAS summary statistics for psoriasis are also available, for which 63 risk loci have been identified [16]. This work has provided powerful data with which to perform MR analyses [12].

In this study, we first reviewed the literature reporting observational evidence for associations between BMI and psoriasis and extended the observational associations in two large, population-based studies. We then applied MR to test for evidence of causality, strength of association, and the direction of causality between BMI and psoriasis.

## Methods

### Literature review and meta-analysis

We searched for published studies that compare the weight or overweight or obesity rates between individuals with psoriasis and healthy controls. All studies identified in a PubMed search were considered for review. PubMed was searched on July 8, 2016 with the terms “psoriasis AND (obesity OR overweight OR BMI).” The inclusion criteria were as follows: an operationalized definition of psoriasis (any definition was accepted, including psoriatic arthritis), inclusion of cases meeting this definition of psoriasis plus a control group without psoriasis, and presentation of data for a BMI-related trait within the psoriasis and control groups. Studies were excluded if they did not present data for both groups, if they did not present usable data in the paper, if they matched individuals with psoriasis and controls on BMI, or if cases and controls were both drawn from a disease subpopulation. We did not exclude studies in which participants may have incidental comorbidities. We extracted the location of the study, the study name (if applicable), age of the study population, features of the control group (e.g., if participants were drawn from another dermatological population), the type of study, how psoriasis was determined (if it was current, recent, or lifetime disease), the covariates used in the analysis, and the definition of overweight and obesity used by the study. All data pertaining to weight and psoriasis were extracted, and a meta-analysis was performed of the definition with the most available data (mean difference in BMI between cases and controls). The following formula was used to obtain an approximate odds ratio (OR) of the effect of BMI on psoriasis, as previously demonstrated by Perry and colleagues [17]:

OR=exp(1.81×[SD×SMD])

such that SD is the standard deviation increase in BMI per SD change in the BMI genetic instrument (genetic risk score [GRS]), SMD is the standardized mean difference, and 1.81 is the scaling factor used to convert SMDs to ln(ORs) [18,19] (S1 Appendix Supporting Text A).

The meta-analysis was conducted separately for children and adults, as well as combined. A random-effects model was used due to the inclusion of heterogeneous populations and study designs being meta-analyzed. Egger regression was also performed to detect the presence of publication bias.

### Investigating causal relationships

#### Study populations

Data were available for a total of 396,495 participants, including 5,676 psoriasis cases from the UK Biobank, aged between 40 and 69 years [20], and 1,076 psoriasis cases from the third survey of the Nord-Trøndelag Health Study (HUNT; 2006–2008), aged 20 years and over [21] (Table 1). All individuals included were of European ancestry and had provided written informed consent. UK Biobank has received ethics approval from the National Health Service National Research Ethics Service (reference 11/NW/0382; UK Biobank application number 10074). The HUNT Study was approved by the Regional Committee for Medical and Health Research Ethics (REC Central). Approval was also received from the Regional Committee for Medical and Health Research Ethics in Mid-Norway (2015/586, 2015/2003).

**Table 1:** Descriptive statistics of data sets used in the study.

Data set	Sample size	Psoriasis cases/controls (% of cases)	Females (%)	Mean (SD) age (years)	Mean (SD) BMI (kg/m^2^)
**UK Biobank**	378,274	5,676/372,598 (1.5%)	203,912 (53.9%)	57.2 (8.0)	27.4 (4.8)
**HUNT**	18,221	1,076/17,145 (5.8%)	10,076 (55.3%)	53.7 (15.2)	27.2 (4.4)
**BMI GWAS** [14]	322,154	**-**	**-**	**-**	27.1 (4.6)
**Psoriasis GWAS** [16]	34,772	13,229/21,543 (38.0%)	**-**	**-**	**-**

**Abbreviations**: BMI, body mass index; GWAS, genome-wide association study; HUNT, the Nord-Trøndelag Health Study; SD, standard deviation.

Summary-level data were also available for 356,926 individuals of European ancestry from published GWASs for BMI [14] (*n* = 322,154) and psoriasis [16] (*n* = 34,772).

#### Clinical outcomes

The BMI of UK Biobank participants was calculated from standing height and weight measurements that were taken while visiting an assessment center. Units of BMI are kilograms per meter squared. Individuals were defined as having psoriasis based on their response during a verbal interview with a trained member of staff at the assessment center. Participants were asked to tell the interviewer which serious illnesses or disabilities they had been diagnosed with by a doctor and were defined as psoriasis cases if this disease was mentioned. Disease information was also obtained from the Hospital Episode Statistics (HES) data extract service in which health-related outcomes had been defined by International Classification of Diseases (ICD)-10 codes (S1 Appendix Table A).

Within HUNT, participants’ height and weight were measured and used to calculate BMI (kg/m^2^). Participants were defined as psoriasis cases based on their response to a general questionnaire sent to all HUNT participants. Psoriasis cases were identified by affirmative response to the question “Have you had or do you have psoriasis?” The diagnostic properties of the psoriasis question have been validated in HUNT (positive predictive value was 78%; 95% CI 69–85) [22].

#### Genotyping

Genotyping of UK Biobank participants was performed with one of two arrays (The Applied Biosystems UK BiLEVE Axiom Array [Affymetrix] and Applied Biosystems UK Biobank Axiom Array). Sample quality control (QC) measures included removing individuals who were duplicated and highly related (third degree or closer), had sex mismatches, or those identified to be outliers of heterozygosity and of non-European descent. Further details of the QC measures applied and imputation performed have been described previously [23–26].

Genotyping of the HUNT participants was performed with one of three different Illumina HumanCoreExome arrays (HumanCoreExome12 version 1.0, HumanCoreExome12 version 1.1, and UM HUNT Biobank version 1.0). The genotypes from different arrays had QC performed separately and were reduced to a common set of variants across all arrays. Sample QC measures were similar to those applied to the UK Biobank. Related individuals were excluded from the analysis (*n* = 30,256). Details of the genotyping, QC measures applied, and imputation have been described elsewhere [27].

#### Confounder variables

Within UK Biobank, confounders that were considered in the current study were age, sex, smoking status, alcohol intake, and educational attainment. The age and sex of participants were baseline characteristics determined at recruitment. The information on age was coded and analyzed as a continuous variable, while sex was analyzed as a binary variable.

Smoking status, alcohol intake, and educational attainment were defined by responses to a touchscreen questionnaire. The smoking status of participants was summarized as being a current, previous, or never smoker, and this information was coded into a categorical variable. Alcohol intake frequency was determined by asking participants “About how often do you drink alcohol?” for which options included “Daily or almost daily,” “Three or four times a week,” “One to three times a month,” “Special occasions only,” and “Never.” This information was categorized for daily, weekly, and monthly alcohol intake. Educational attainment was also defined by asking “Which of the following qualifications do you have?” for which participants could select more than one option, including “College or University degree,” “A levels/AS levels or equivalent,” “O levels/GCSEs or equivalent,” “CESs or equivalent,” “NVQ or HND or HNC or equivalent,” “Other professional qualifications, e.g., nursing, teaching,” or “None of the above.” Participant responses were coded into categorical variables for degree holders, as well as for those who had completed advanced-level studies (A-level) or had obtained their general certificate of secondary education (GCSE).

Within HUNT, confounders considered in the current study were age, sex, smoking status, and alcohol intake. Information on educational attainment was not available in the third survey of the HUNT Study. The age and sex of participants were determined at the time of participation. The information on age was coded and analyzed as a continuous variable, while sex was analyzed as a binary variable.

Smoking status and alcohol intake were defined by the participants’ response to a questionnaire. Smoking status was defined as being never, former, occasional, or current smoker. Alcohol intake frequency was determined by asking participants “About how often in the last 12 months did you drink alcohol?” for which options included “4–7 times a week,” “2–3 times a week,” “about once a month,” “a few times a year,” “not at all last year,” and “never drunk alcohol.”

#### Observational analysis

Within the UK Biobank and HUNT data sets, logistic regression models were used to estimate the observational association between BMI and psoriasis. Analyses were adjusted for age, sex, smoking status, alcohol intake, and educational attainment (where information was available in UK Biobank only). The estimates for each data set were meta-analyzed assuming a fixed-effect model.

#### Defining genetic instruments

The genetic instrument for BMI comprised the 97 BMI-associated single-nucleotide polymorphisms (SNPs) reported by the GIANT consortium to account for approximately 2.7% of BMI variation (a meta-analysis of 125 GWASs with 339,224 individuals) [14]. These SNPs were extracted from both the UK Biobank and HUNT data sets (S1 Appendix Table B and C) to perform one-sample MR analysis in each data set. We also combined these SNPs to create a standardized GRS using the --score command in PLINK (version 1.9). In doing so, the dosage of the effect allele for each SNP was weighted by the effect estimates reported for the European sex-combined analysis (*n* = 322,154) by Locke and colleagues [14], summed across all variants, and divided by the total number of variants. The scores were standardized to have a mean of 0 and an SD of 1.

The BMI-associated SNP rs12016871 was not present within the UK Biobank and HUNT data sets, therefore rs9581854 was used as a highly correlated proxy (r^2^ = 1.0) (S1 Appendix Table D).

The BMI-associated SNPs most recently reported by Yengo and colleagues [15] were also used as an updated genetic instrument for BMI.

For the psoriasis genetic instrument, 62 psoriasis-associated SNPs (outside of the human leukocyte antigen [HLA] region) were obtained from the most recent psoriasis GWAS (a meta-analysis of 13,229 cases and 21,543 controls of European ancestry) [16]. These SNPs were extracted from both the UK Biobank and HUNT data sets and were used as instruments to perform one-sample MR analysis in each data set. These SNPs were also combined to create a standardized GRS, in which they were weighted by their published effect sizes. The psoriasis-associated SNP rs118086960 was not present in the UK Biobank or HUNT data sets and had no suitable proxy (r^2^ > 0.8). Therefore, 61 independent SNP associations were used as a genetic instrument to perform the one-sample MR analysis (S1 Appendix Table E and F).

The reported BMI-associated SNPs and psoriasis-associated SNPs were also used to perform two-sample MR analysis, using summary data from the published GWAS for each trait [14,16].

#### MR analysis

**Fig 1:**
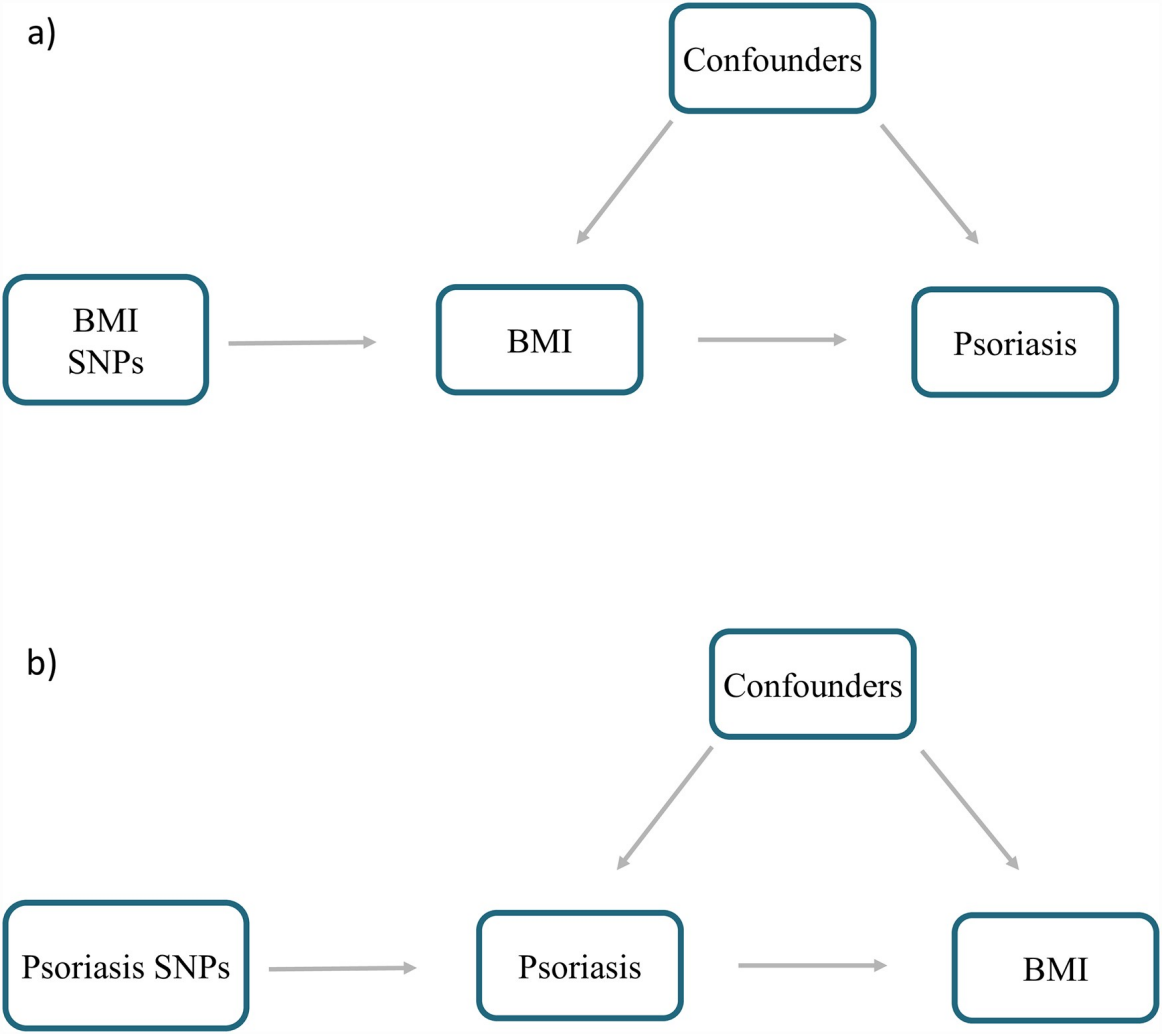
Schematic representation of MR analyses. (a) BMI SNPs were used as instrumental variables to investigate the causal effect of BMI upon psoriasis. (b) Psoriasis SNPs were used as instrumental variables to investigate the causal effect of genetic risk of psoriasis upon BMI. Arrows indicate MR assumption such that the instrumental variable is associated with the exposure—not associated with confounders—and only affects the outcome via the exposure. BMI, body mass index; MR, mendelian randomization; SNP, single-nucleotide polymorphism.

One-sample MR analysis was performed separately in UK Biobank as well as the HUNT data set, using individual-level data with participants’ BMI SNPs, measured BMI, and disease outcome status (Fig 1). The MR estimates from each genetic instrument (SNP) were meta-analyzed assuming a random-effects model, giving a single estimate for the analysis performed in each data set. A random-effects model was used here, to avoid overprecision of the causal estimate and to allow for heterogeneity in the causal estimates being meta-analyzed from the different genetic variants.

The MR analysis with the individual BMI SNPs was performed with the two-stage predictor substitution (TSPS) method [28]. The first stage involved regression of BMI upon individual BMI SNPs. The outcome (psoriasis) was then regressed upon the fitted values from the first regression stage. Because psoriasis is a binary outcome, the first-stage linear regression was restricted to individuals who were controls for psoriasis only, as recommended by Burgess and colleagues [29]. Logistic regression was then performed in the second stage, in which the fitted values for the cases were predicted. The standard errors (SEs) of these estimates were adjusted using the first term of the delta method expansion for the variance of a ratio, allowing for the uncertainty in the first regression stage to be taken into account [29].

Genetic principal components (as previously described [25–27]) were included as covariates in the analysis to control for residual population structure. The UK Biobank analysis also controlled for the platform used to genotype the samples. In the HUNT data set, the genotyped data were reduced to a common set of variants across all platforms before imputation.

Two-sample MR analysis of published GWAS data was performed using the “MendelianRandomisation” R package [30,31]. Estimates for the association between BMI and BMI SNPs in Europeans were taken from the GIANT BMI GWAS published by Locke and colleagues [14]. Summary statistics from the most recent psoriasis [16] GWASs were used to obtain estimates for the association of psoriasis with the BMI SNPs in Europeans. The published BMI SNP estimates were based on an inverse normal transformation of BMI residuals on age and age squared, as well as any necessary study-specific covariates. In unrelated individuals, residuals were calculated according to sex and case/control status and were sex-adjusted among related individuals [14]. Therefore the causal estimates for the two-sample analysis were converted to raw BMI units (kg/m^2^), assuming a median BMI SD of 4.6 kg/m^2^ [14].

The one- and two-sample estimates were meta-analyzed assuming a fixed-effect model to obtain an overall causal estimate, assuming no between-method heterogeneity.

An additional two-sample MR analysis was performed in the same manner, using BMI SNP–BMI association estimates from the more recent BMI meta-analysis by Yengo and colleagues, in which 716 SNPs had been reported to account for approximately 5% of the variance of BMI [15].

#### Sensitivity analysis

MR-Egger regression, weighted median analysis, and the weighted mode-based estimate (MBE) were used to investigate potential pleiotropy. SNPs that act through a pleiotropic pathway would violate the MR assumption that the instrumental variable has an effect upon the outcome only via the exposure being investigated and could bias the causal estimate. The weighted median method provides a valid causal estimate if at least 50% of the information each instrument contributes to the analysis comes from valid instruments [32]. Likewise, the weighted MBE also provides a valid causal estimate if the largest weights are from valid instruments [33], while the intercept from the MR-Egger regression analysis allows the size of any pleiotropic effect to be determined [34]. MR-Egger regression gives a valid causal estimate under the InSIDE assumption, in which each SNP–exposure association is independent of the direct pleiotropic effect of the SNP [34].

In addition, one-sample MR analysis was performed using the *FTO* SNP alone (rs1558902) as a genetic instrument due to its strong association with BMI [35].

Because the instrumental variables used in an MR analysis are assumed to be independent of confounders, we investigated the relationship between the BMI GRS and potential confounders of BMI by performing a simple regression of the confounder upon the BMI GRS. The relationship between the *FTO* variant and potential confounders was also investigated.

#### Reverse-direction MR analysis

We also investigated the causal effect of the genetic liability of psoriasis upon BMI (Fig 1). One-sample MR analysis was performed in UK Biobank and also in the HUNT data set with the two-staged least squares (TSLS) method, in which psoriasis-associated SNPs were used as instruments. As with the TSPS method, this analysis involves two regression stages. Psoriasis was regressed upon the psoriasis genetic instrument; the outcome (BMI) was then regressed upon the fitted values from the first stage regression. The one-sample MR estimates from each data set were then meta-analyzed assuming a fixed-effect model to give a single causal estimate (change in BMI per log odds of psoriasis). In addition, one-sample MR analysis was also performed such that the exposure (genetic liability of psoriasis) was considered a linear variable with values from 0 to 1 to aid interpretation of the causal estimate (difference in BMI between psoriasis cases and controls) (S1 Appendix Supporting Text B). Two-sample MR analysis was also performed using the “MendelianRandomisation” R package [31] with summary results from GWASs for psoriasis [16] and from the GIANT BMI GWAS [14]. The one- and two-sample MR estimates were meta-analyzed using a fixed-effect model to give a final causal estimate. For the sake of interpretation, the estimates obtained were multiplied by 0.693 to represent the change in BMI per doubling in odds of psoriasis, as demonstrated by Gage and colleagues [36].

Sensitivity analyses were performed with MR-Egger regression, weighted median, and weighted MBE methods. A separate two-sample MR analysis was performed in the same manner, such that psoriasis SNP–BMI association estimates were extracted from the more recent BMI meta-analysis [15].

Variants within the HLA region were not included in the genetic instrument due to the pleiotropic nature of the region. However, two-sample MR analysis was performed using the SNP rs13200483 alone as an instrument that tags the HLA-C*06:02 allele and is strongly associated with psoriasis [37]. SNP estimates were taken from the most recent psoriasis GWAS [16] and the GIANT BMI study published by Locke and colleagues [14].

All analyses were performed using R (www.r-project.org) unless otherwise stated. There was no formal prespecified protocol for this study. The main analyses and sensitivity analyses described above were decided on beforehand. This is with the exception of the sensitivity analysis performed with the most recent BMI meta-analysis [15], in order to demonstrate use of the most current GWAS summary data for BMI. We also performed sensitivity analysis with a variant at the HLA-C*06:02 locus as recommended by the reviewers. One-sample MR analyses were also performed within UK Biobank, stratifying for psoriasis individuals who were self-reported, or defined by the HES data extract service in response to reviewers’ comments. Furthermore, publication bias was investigated for the meta-analysis of previously reported studies for the relationship between BMI and psoriasis as suggested by the reviewers.

## Results

### Literature review and meta-analysis

**Fig 2:**
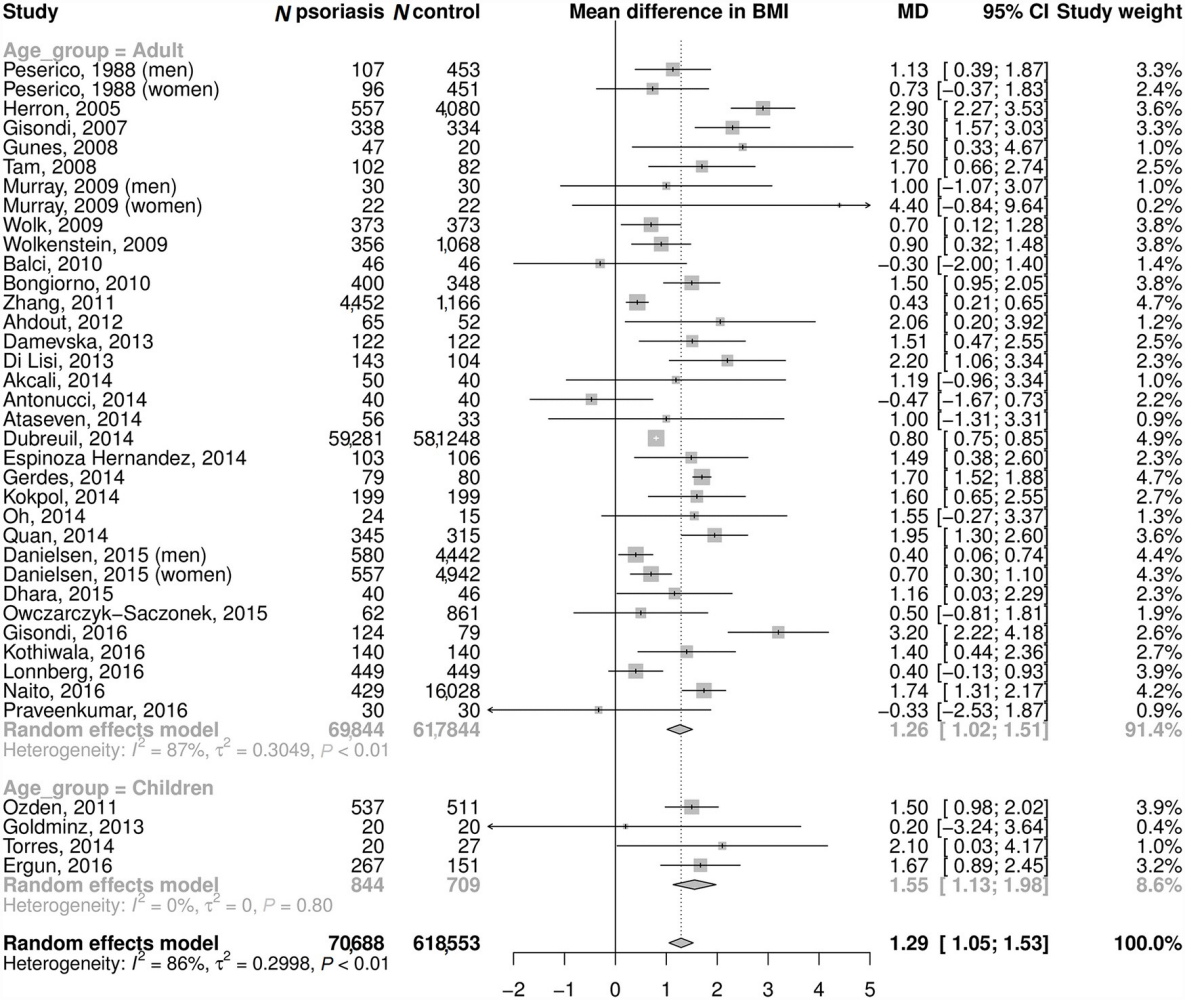
Observational association between BMI and psoriasis. Meta-analysis of mean difference in BMI (kg/m^2^) between psoriasis cases and controls. MD of 1.26 kg/m^2^ in adults is equivalent to OR of 1.092. MD of 1.55 kg/m^2^ in children is equivalent to OR of 1.093. BMI, body mass index; MD, mean difference; OR, odds ratio.

We identified 56 studies reporting data on the relationship between psoriasis and BMI, obesity, or being overweight (see Fig A in S1 Appendix). A total of 35 studies that compared mean BMI between psoriasis cases and controls (Fig 2; S1 Appendix Supporting Text C) were taken forward to be meta-analyzed. The meta-analysis found a mean difference in BMI between psoriasis cases and controls of 1.26 kg/m^2^ (95% CI 1.02–1.51) among adults (69,844 psoriasis cases and 617,844 controls) and 1.55 kg/m^2^ (95% CI 1.13–1.98) in children (844 psoriasis cases and 709 controls). The ages of pediatric psoriasis patients ranged from 5 to 18 years. Where stated, the majority of the studies had defined adults to be those aged 18 years and older. However, a number of studies had used the age threshold of 15 years [38–42], and one used 17 years to define adulthood [43]. For both adults and children, the observed difference in BMI was equivalent to a 9% increase in the odds of psoriasis per 1 kg/m^2^ increase in BMI. Twenty-one other studies tested for an association between BMI or obesity traits and psoriasis using alternative models (S1 Appendix Table G). These all reported a positive association, including two studies that reported the odds of psoriasis in adults per 1 kg/m^2^ increase in BMI to be 1.09 (95% CI 1.04–1.16) [43] and 1.04 (95% CI 1.02–1.10) [38]. We detected very little evidence of publication bias in the meta-analysis (S1 Appendix Fig B).

### Genetic instruments

The BMI GRS was strongly associated with BMI in UK Biobank (Beta = 0.64; 95% CI 0.63–0.66, F-statistic = 7,091, R^2^ = 1.8%) and HUNT (Beta = 0.66; 95% CI 0.60–0.72, F-statistic = 422, R^2^ = 2.3%) (S1 Appendix Fig C and D), providing evidence in support of the strength of this instrument. We investigated the association between the BMI GRS and potential confounders of BMI. Some small effects on the confounders were seen; however, the strength of association was minimal in comparison to the association with BMI. This was also true for the *FTO* variant alone, which is unlikely to have horizontal pleiotropic effects on these confounders (S1 Appendix Fig E and F). The GRS derived for psoriasis was a good predictor of psoriasis in the UK Biobank (OR = 1.55; 95% CI 1.51–1.59, F-statistic = 6,415, R^2^ = 2.1%) and HUNT (OR = 1.41; 95% CI 1.33–1.50, F-statistic = 340, R^2^ = 1.8%) data sets.

### Effect of BMI upon psoriasis

#### Observational analysis

**Fig 3:**
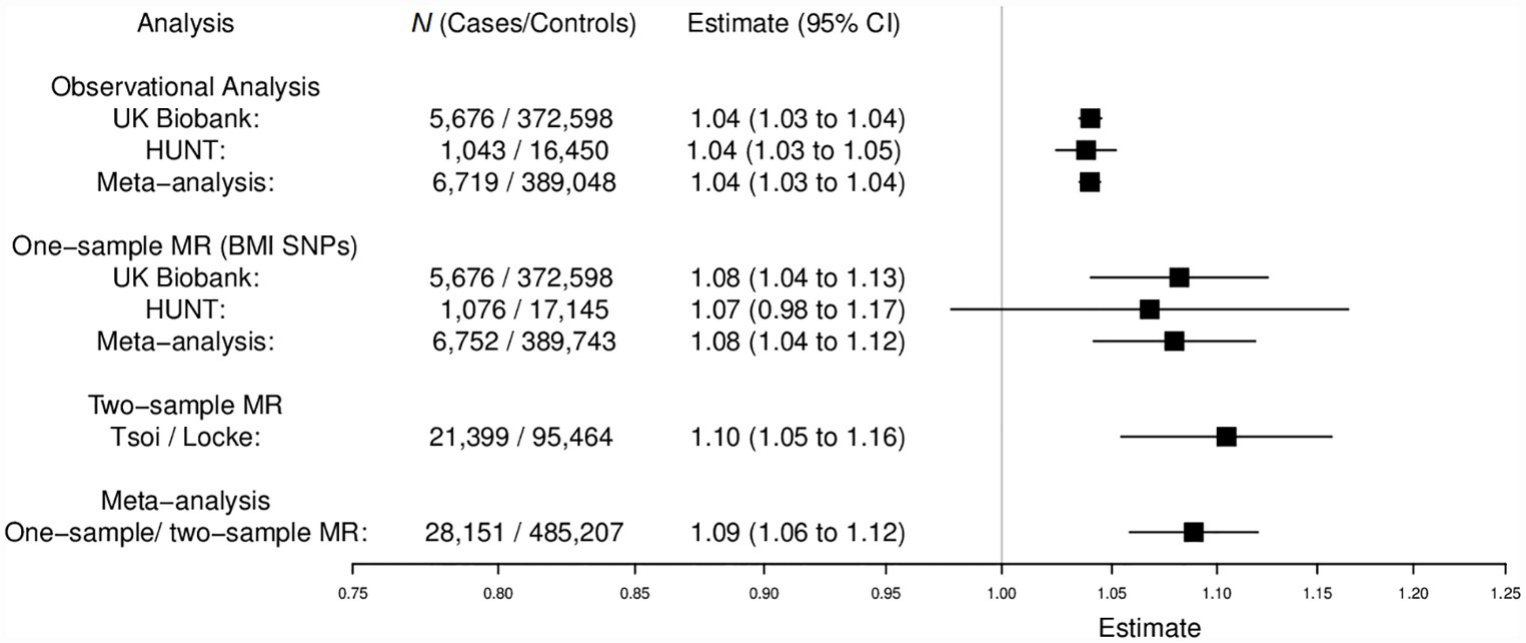
Effect of BMI upon psoriasis. Meta-analysis of observational and one-sample and two-sample MR causal estimates (using individual BMI SNPs as instrumental variables). Observational analysis in HUNT is restricted to individuals with complete information on potential confounders. One-sample MR was performed separately in UK Biobank and HUNT using individual-level data. Two-sample MR was performed with published GWAS summary-level data for BMI [14] and psoriasis [16]. Estimates are given as change in odds per 1 kg/m^2^ increase in BMI. BMI, body mass index; CI, confidence interval; GWAS, genome-wide association study; HUNT, the Nord-Trøndelag Health Study; MR, mendelian randomization; SNP, single-nucleotide polymorphism.

Higher BMI was associated with increased risk of psoriasis in both the UK Biobank and HUNT data sets. Overall, a 1 kg/m^2^ increase in BMI was associated with 4% higher odds of psoriasis (meta-analysis OR = 1.04; 95% CI 1.03–1.04; *P* = 1.73 × 10^−60^) (Fig 3), slightly lower than that estimated from published literature.

#### MR

MR performed with UK Biobank, HUNT, and published GWAS data gave evidence that higher BMI increases the risk of psoriasis. The causal estimate from UK Biobank showed about an 8% increase in odds of psoriasis per 1 kg/m^2^ higher BMI (OR = 1.08; 95% CI 1.04–1.13; *P* = 8.75 × 10^−5^). Similar causal estimates were also found when stratifying by psoriasis cases that were self-reported and those defined with the HES data extract service (S1 Appendix Table H). In HUNT, about a 7% increase was shown (OR = 1.07; 95% CI 0.98–1.17; *P* = 0.14). The two-sample estimate from published GWAS data [14] also provided evidence of higher psoriasis risk with increased BMI (OR = 1.10, 95% CI 1.05–1.16; *P* = 6.46 × 10^−5^) (Fig 3). Meta-analysis of both one-sample and two-sample estimates produced an overall causal estimate of 1.09 per 1 kg/m^2^ higher BMI (95% CI 1.06–1.12; *P* = 4.67 × 10^−9^, *I*^2^ statistic = 0.0%) (Fig 3), consistent with the observational estimate from our meta-analysis of the published literature. This estimate suggests that, for example, an increase in BMI of 5 units from 25 to 30 would increase the risk of psoriasis by 53% (OR per 5-unit higher BMI = exp[Beta per 1-unit higher BMI × 5]).

There was little evidence of pleiotropy in the MR-Egger regression analysis (UK Biobank intercept = 0.00; 95% CI −0.01 to 0.01; *P* = 0.63, HUNT intercept = 0.00; 95% CI −0.02 to 0.02; *P* = 0.96), and the sensitivity analyses all gave similar estimates (S1 Appendix Fig G and Table I). In addition, when limiting the instrument to only the *FTO* SNP, a similar estimate (although with a wider CI) was observed (OR = 1.11; 95% CI 1.04–1.19; *P* = 1.22 × 10^−3^) (S1 Appendix Fig H).

Two-sample MR analysis was also performed using the most recent, larger number of BMI SNP estimates [15]. This gave a similar estimate to the overall causal estimate obtained (OR = 1.10; 95% CI 1.06–1.13; *P* = 1.59 × 10^−4^).

### Reverse MR analysis: Genetic liability for psoriasis upon BMI

**Fig 4:**
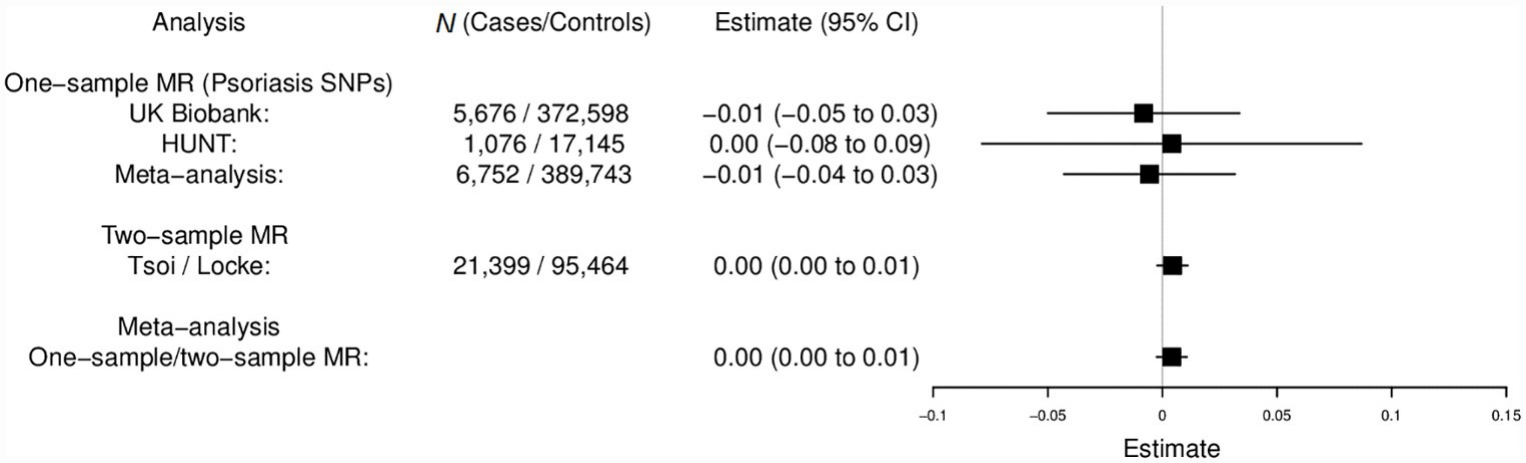
Reverse-direction MR analysis: Genetic liability for psoriasis upon BMI. Meta-analysis of one-sample and two-sample MR estimates (using individual psoriasis SNPs as instrumental variables). One-sample MR was performed separately in UK Biobank and HUNT using individual-level data. Two-sample MR was performed with published GWAS sum mary-level data for BMI [14] and psoriasis [16] Estimates represent the change in BMI (kg/m^2^) per doubling odds of psoriasis. F-statistic = 6,415, R^2^ = 2.1% (UK Biobank); F-statistic = 340, R^2^ = 1.8% (HUNT). BMI, body mass index; CI, confidence interval; GWAS, genome-wide association study; HUNT, the Nord-Trøndelag Health Study; MR, mendelian randomization; SNP, single-nucleotide polymorphism.

The meta-analysis of UK Biobank, HUNT, and the two-sample data found no strong evidence for a causal effect of the genetic risk of psoriasis on BMI (0.004 kg/m^2^ change in BMI per doubling odds of psoriasis, 95% CI −0.003 to 0.011, *P* = 0.23) (Fig 4). Similarly, no strong evidence of a causal effect was found when performing two-sample MR analysis with the variant at HLA-C*06:02 (rs13200483) alone, (0.03 kg/m^2^ change in BMI per doubling odds of psoriasis, 95% CI −0.02 to 0.07, *P* = 0.24). Such estimates may be prone to misinterpretation if there is any heterogeneity of the effect within different subpopulations (e.g., effect only in a subset of the population, such as those with psoriasis) [44]. However, we found no strong evidence for such heterogeneity when comparing BMI variance across levels of the psoriasis genetic score in UK Biobank. The meta-analysis of the one-sample UK Biobank and HUNT estimates also estimated the increase in BMI in psoriasis cases compared to controls to be 0.27 kg/m^2^ (95% CI −2.02 to 2.55) by treating psoriasis as a linear variable in the analysis. Because this estimate is much smaller than the observational estimate (1.26 kg/m^2^) and the forward-direction MR estimate is very consistent with the observational estimate, we conclude that the majority of the relationship is due to a causal effect of BMI on psoriasis, rather than the other way around.

## Discussion

The rising prevalence of psoriasis and obesity are important public health concerns [3,5,37,45]. We found evidence of increased BMI having a causal effect upon occurrence of psoriasis, and the estimated effect size is of a magnitude that is likely to be clinically significant (9% increased risk of psoriasis for 1 unit increase in BMI). Furthermore, the direction and magnitude of effect seen is notably consistent with that seen observationally and in previous literature. In the reverse direction, the estimate of 0.27 kg/m^2^ suggests much less influence of psoriasis on an individual’s BMI. Overall, our results give evidence that the observational estimates in the literature are predominantly explained by a causal effect of BMI upon psoriasis and are not substantially impacted by unmeasured confounding, implying that excess adiposity is part of the reason for some individuals developing psoriasis.

A key limitation of MR analysis is the possibility of pleiotropic mechanisms (from genetic instrument to outcome, not via the exposure) invalidating the method. We performed various sensitivity analyses to explore potential pleiotropic effects of the SNPs that make up the BMI instrument. When restricting the instrument to only the *FTO* variant, for which there is good understanding of the biological mechanism [35], we found the estimate from this analysis to be consistent with the estimate using all BMI SNPs. This suggests that the causal estimates seen are not predominantly driven by pleiotropic SNPs with alternative biological effects. This is supported by the MR-Egger regression intercepts that were centered around zero (indicating no directional pleiotropy among the included variants).

Our analysis has included a total of 753,421 individuals, including data from two of the biggest population-based studies currently available, and one of the largest published GWASs. We have applied both one-sample and two-sample MR approaches, and the estimates from these analyses were meta-analyzed to provide increased statistical power. The use of a strong genetic instrument for BMI provides an additional strength to this study.

There are some likely limitations to this study. The data in our study included contemporaneous measurements of BMI but relied predominantly on patient report or recall for the ascertainment of psoriasis. This disease may follow an acute or chronic relapsing and remitting course. We do acknowledge that the possible misdiagnosis of psoriasis, and mild sufferers remaining undiagnosed, should be taken into account when interpreting the results of this study, especially given the little overlap of psoriasis cases who are self-reported and also defined with the HES data extract service in UK Biobank (S1 Appendix Table H). Our MR estimate is also limited in that it only informs on the lifetime impact of higher BMI on psoriasis, rather than the possible effect of a short-term intervention aiming to reduce BMI in clinical practice. Furthermore, because the cohorts studied were of European ancestry, the ethnic variation of participants in the study is limited. In addition, the BMI SNPs used are a stronger instrument for adult BMI compared to childhood BMI [46]. In further work, the separate effects of visceral and subcutaneous fat may also be considered because these are likely to have a greater impact upon inflammation compared to BMI alone.

Despite the large sample sizes included in the current study, the one-sample estimates still have wide CIs (due to the relative low number of cases in population-based cohorts). Nonetheless, the similarity of the causal estimates found when analyzing UK Biobank, HUNT, and previous GWAS data does increase confidence in our findings. As expected with a large sample size, we did observe some associations between the BMI GRS and potential confounders of BMI. However, we found these to be minimal in comparison to the strength of the association with BMI and therefore unlikely to be materially affecting the results. Nevertheless, it is important to note the possible influences of unmeasured confounders, especially when utilizing large data resources such as the UK Biobank.

There are various possible mechanisms linking obesity with skin inflammation due to functional changes within adipose tissue as well as quantitative effects, such as the increased production of inflammatory cytokines from adipose tissue [47]. Excess skin adipose tissue results in pro-inflammatory cytokine and hormone secretion. Cytokines such as tumor necrosis factor alpha (TNFα) and interleukin 6 (IL-6) are directly implicated in the pathology of psoriasis and are targets for some highly effective treatments [48,49]. Leptin can increase keratinocyte proliferation and pro-inflammatory protein secretion, which are characteristics of psoriasis [50], while the secretion of adiponectin, which is putatively anti-inflammatory [10], is reduced in the obese state. The skin of obese individuals shows features of impaired barrier function [51], while impairment in lymphatic function may delay the clearance of inflammatory mediators [47]. Other mechanisms remain possible; however, these are weakly researched. Our results, supporting a causal relationship, suggest that this area warrants further detailed work.

Our findings suggest that approaches to the prevention and treatment of psoriasis might come from targeting adiposity levels in addition to the immune pathways in skin. Although our results imply that such interventions may be effective in the prevention of psoriasis, they cannot determine that they would be effective at improving the disease course after onset. However, our findings do suggest that this is a promising area to explore, particularly with validation in a clinical trial setting to determine what magnitude of effect a particular intervention may have. This is also supported by previous reports of weight loss improving psoriatic skin and joint disease [52–54]. The concept of managing cardiovascular risk factors is already included in clinical guidelines for psoriasis, where—although a strong observational relationship has been found between these two traits [55]—there has so far been little genetic or epigenetic overlap to support this [56]. In comparison, our findings provide evidence of causality for the observational relationship between higher BMI and psoriasis. Furthermore, our data suggest a potential to yield meaningful clinical benefits via a causal effect on skin disease. Our findings come at a time when weight loss strategies are improving in the community, with a variety of evidence-based interventions now emerging [57,58]. We believe that the need for further trials of weight loss at different stages of psoriasis is strengthened by our work. Although it has not been possible to investigate in the current study, analyzing the causal effect of BMI upon severe psoriasis or various disease subtypes will also be of clinical value. We also note that the potential health implications of this study will be dependent upon elevated BMI in the community being amenable to intervention.

In conclusion, our findings indicate a causal effect of BMI upon psoriasis, which carries possible health implications. These results provide further evidence supporting the need to effectively manage obesity in the general population as well as in patients with psoriasis.

## Supporting information

S1 Appendix Supporting text, figures, and tables. (PDF)

S1 STROBE Checklist STROBE, STrengthening the reporting of OBservational studies in Epidemiology. (DOC)

## Abbreviations

BMI: body mass index
GCSE: general certificate of secondary education
GIANT: Genetic Investigation of Anthropometric Traits
GRS: genetic risk score
GWAS: genome-wide association study
HES: Hospital Episode Statistics
HLA: human leukocyte antigen
HUNT: the Nord-Trøndelag Health Study
ICD: International Classification of Diseases
IL-6: interleukin 6
MBE: mode-based estimate
MR: mendelian randomization
OR: odds ratio
QC: quality control
SD: standard deviation
SE: standard error
SMD: standardized mean difference
SNP: single-nucleotide polymorphism
TNFα: tumor necrosis factor alpha
TSLS: two-staged least squares
TSPS: two-stage predictor substitution
