# Supplementary material for: Evidence of a causal relationship between body mass index and psoriasis: A mendelian randomization study
Source: PLoS Med. 2019 Jan 31;16(1):e1002739. doi: 10.1371/journal.pmed.1002739 (PMC6354959; doi:10.1371/journal.pmed.1002739)
Supplement: S1 Appendix — (PDF) [file pmed.1002739.s001.pdf]

S1 Appendix

Evidence of a causal relationship between body mass index and psoriasis: a Mendelian Randomization study

Budu-Aggrey A et al

Supporting Text

Supporting Text A. Conversion of mean difference to odds ratios

As demonstrated by Perry *et al* [1], an effect estimate such as the standardised mean difference (SMD) can be converted to an odds ratio using the following formula:

$$\boldsymbol{OR = \exp(1.81\ x\ (SD\ x\ SMD))}$$

In support of this, Borenstein *et al* [2] stated that the SMD can be converted to the log odds ratio with the following formula:

$$\boldsymbol{log\ odds\ ratio = SMD\ x\ \frac{\pi}{\sqrt{3}}}$$

The method assumes continuous data to have a logistic distribution rather a normal distribution. As analyses of continuous outcomes are commonly performed assuming a normal distribution, the variance (V) of the log odds ratio is as follows:

$$\boldsymbol{V_{LogOddsRatio} = V_{SMD}\ x\ \frac{\pi^2}{3}}$$

The variance of the standard logistic distribution is $\pi^2/3$, therefore the SMD can be converted to ln(odds) by multiplying by $\pi/\sqrt{3}$ or 1.81 to 2 decimal places [3].

This method was applied in the current study to obtain odds ratios for the association between BMI and psoriasis found during the meta-analysis of previously reported observational studies. In applying the first formula, SD denotes the standard deviation change in BMI per standard deviation change in the BMI genetic instrument. In individuals from the UK Biobank, we found this to be 0.135, using the BMI genetic risk score as the genetic instrument. SMD denotes the standardised mean difference in BMI estimated from the association studies that were meta-analysed. In adults this was found to be 0.360 (95% CI = 0.206, 0.515), and 0.363 in children (95% CI = 0.262, 0.465).

1. Perry JRB, Weedon MN, Langenberg C, Jackson AU, Lyssenko V, Sparsø T, et al. Genetic evidence that raised sex hormone binding globulin (SHBG) levels reduce the risk of type 2 diabetes. Hum Mol Genet. Oxford University Press; 2010;19: 535–544. doi:10.1093/hmg/ddp522

2. Borenstein M, Hedges L, Rothstein H. Converting Among Effect Sizes. Introduction to Meta-Analysis. Chichester, UK: John Wiley & Sons, Ltd; 2009. pp. 45–49. doi:10.1002/9780470743386.ch7

3. Chinn S. A simple method for converting an odds ratio to effect size for use in meta-analysis. Stat Med Stat Med. 2000;19: 3127–3131. Available: http://www.aliquote.org/pub/odds_meta.pdf

Supporting Text B. Reverse direction MR analysis: genetic liability as a linear variable

An additional one-sample MR analysis was performed where the genetic liability of psoriasis was considered as a linear variable with values from "0" to "1". This was performed in the UK Biobank and HUNT datasets using the two-staged least squares (TSLS) method with the "ivpack" R package [1] using individual SNPs for psoriasis as an instrument. This analysis involves two linear regression stages where psoriasis is first regressed upon the instrument (disease-associated SNPs), then the outcome (BMI) is regressed upon the fitted values from the first stage regression. The estimates from each genetic instrument were meta-analysed assuming a random effects model to give a single estimate for the effect of psoriasis genetic risk upon BMI within UK Biobank and HUNT. The estimates from each dataset were then meta-analysed assuming a fixed effect model to give an overall causal effect.

1. Baiocchi M, Cheng J, Small DS. Instrumental variable methods for causal inference. Stat Med. 2014;33: 2297–2340. doi:10.1002/sim.6128

Supporting Text C. Reviewed studies reporting a mean difference in BMI between psoriasis cases and controls

<u>Adult psoriasis studies</u>

Peserico A, Zanetti G, Padovan S, Bertoli P, Veller Fornasa C, Cipriani R, et al. Relationship between body weight and blood pressure and some metabolic parameters in psoriatic patients. Br J Dermatol. 1988;118: 191–194. Available: https://onlinelibrary.wiley.com/doi/pdf/10.1111/j.1365-2133.1988.tb01773.x

Herron MD, Hinckley M, Hoffman MS, Papenfuss J, Hansen CB, Callis KP, et al. Impact of Obesity and Smoking on Psoriasis Presentation and Management. Arch Dermatol. American Medical Association; 2005;141: 1527–1534. doi:10.1001/archderm.141.12.1527

Gisondi P, Tessari G, Conti A, Piaserico S, Schianchi S, Peserico A, et al. Prevalence of metabolic syndrome in patients with psoriasis: a hospital-based case-control study. Br J Dermatol. Wiley/Blackwell (10.1111); 2007;157: 68–73. doi:10.1111/j.1365-2133.2007.07986.x

Gunes Y, Tuncer M, Calka O, Guntekin U, Akdeniz N, Simsek H, et al. Increased frequency of pulmonary hypertension in psoriasis patients. Arch Dermatol Res. Springer-Verlag; 2008;300: 435–440. doi:10.1007/s00403-008-0859-9

Tam L-S, Tomlinson B, Chu TT-W, Li M, Leung Y-Y, Kwok L-W, et al. Cardiovascular risk profile of patients with psoriatic arthritis compared to controls--the role of inflammation. Rheumatology. Oxford University Press; 2008;47: 718–723. doi:10.1093/rheumatology/ken090

Murray ML, Bergstresser PR, Adams-Huet B, Cohen JB. Relationship of psoriasis severity to obesity using same-gender siblings as controls for obesity. Clin Exp Dermatol. Wiley/Blackwell (10.1111); 2009;34: 140–144. doi:10.1111/j.1365-2230.2008.02791.x

Wolk K, Mallbris L, Larsson P, Rosenblad A, Vingård E, Ståhle M. Excessive body weight and smoking associates with a high risk of onset of plaque psoriasis. Acta Derm Venereol. 2009;89: 492–497. doi:10.2340/00015555-0711

Wolkenstein P, Revuz J, Roujeau JC, Bonnelye G, Grob JJ, Bastuji-Garin S. Psoriasis in France and associated risk factors: Results of a case-control study based on a large community survey. Dermatology. Karger Publishers; 2009;218: 103–109. doi:10.1159/000182258

Balci A, Balci DD, Yonden Z, Korkmaz I, Yenin JZ, Celik E, et al. Increased amount of visceral fat in patients with psoriasis contributes to metabolic syndrome. Dermatology. Karger Publishers; 2010;220: 32–7. doi:10.1159/000254482

Bongiorno M, Doukaki S, Rizzo D, AricÃ2 M. The prevalence of the obesity in patients with moderate to severe psoriasis in Sicily populations. J Eur Acad Dermatology Venereol. Wiley/Blackwell (10.1111); 2010;24: 92–93. doi:10.1111/j.1468-3083.2009.03309.x

Zhang C, Zhu K, Zheng H, Cui Y, Zhou F, Chen Y, et al. The effect of overweight and obesity on psoriasis patients in Chinese Han population: a hospital-based study. J Eur Acad Dermatology Venereol. Wiley/Blackwell (10.1111); 2011;25: 87–91. doi:10.1111/j.1468-3083.2010.03706.x

Ahdout J, Kotlerman J, Elashoff D, Kim J, Chiu MW. Modifiable lifestyle factors associated with metabolic syndrome in patients with psoriasis. Clin Exp Dermatol. Wiley/Blackwell (10.1111); 2012;37: 477–483. doi:10.1111/j.1365-2230.2012.04360.x

Damevska K, Neloska L, Gocev G, Mihova M. Metabolic syndrome in untreated patients with psoriasis: case-control study. JDDG J der Dtsch Dermatologischen Gesellschaft. Wiley/Blackwell (10.1111); 2013;11: 1169–1175. doi:10.1111/ddg.12193

Di Lisi D, Macaione F, Corrado E, Bonura F, Novo G, Peritore A, et al. Cardiovascular risk profile of patients with psoriasis. Recenti Prog Med. 2013;104: 102–5. doi:10.1701/1255.13857

Akcali C, Buyukcelik B, Kirtak N, ˙ Naloz SI. Clinical and laboratory parameters associated with metabolic syndrome in Turkish patients with psoriasis. J Int Med Res. 2014;42: 386–394. doi:10.1177/0300060513502891

Antonucci VA, Tengattini V, Balestri R, Patrizi A, Filippini M, Bardazzi F. Intima-media thickness in an Italian psoriatic population: correlation with lipidic serum levels, PASI and BMI. J Eur Acad Dermatology Venereol. Wiley/Blackwell (10.1111); 2014;28: 512–515. doi:10.1111/jdv.12075

Ataseven A, Kesli R, Kurtipek GS, Ozturk P. Assessment of lipocalin 2, clusterin, soluble tumor necrosis factor receptor-1, interleukin-6, homocysteine, and uric acid levels in patients with psoriasis. Dis Markers. Hindawi; 2014;2014: 541709. doi:10.1155/2014/541709

Dubreuil M, Rho YH, Man A, Zhu Y, Zhang Y, Love TJ, et al. Diabetes incidence in psoriatic arthritis, psoriasis and rheumatoid arthritis: a UK population-based cohort study. Rheumatology. Oxford University Press; 2014;53: 346–352. doi:10.1093/rheumatology/ket343

Espinoza Hernández CJ, Lacy Niebla RM, Soto López ME, Kresch Tronik NS, Vega-Memije ME. Prevalence of metabolic syndrome in patients with psoriasis. Gac Med Mex. 150: 311–6. Available: http://www.ncbi.nlm.nih.gov/pubmed/25098216

Gerdes S, Osadtschy S, Buhles N, Baurecht H, Mrowietz U. Cardiovascular biomarkers in patients with psoriasis. Exp Dermatol. Wiley/Blackwell (10.1111); 2014;23: 322–325. doi:10.1111/exd.12381

Kokpol C, Aekplakorn W, Rajatanavin N. Prevalence and characteristics of metabolic syndrome in South-East Asian psoriatic patients: A case-control study. J Dermatol. 2014;41: 898–902. doi:10.1111/1346-8138.12614

Oh YJ, Lim HK, Choi JH, Lee JW, Kim NI. Serum Leptin and Adiponectin Levels in Korean Patients with Psoriasis. J Korean Med Sci. 2014;29: 729. doi:10.3346/jkms.2014.29.5.729

Quan C, Zhu K-J, Zhang C, Liu Z, Liu H, Zhu C-Y, et al. Combined effects of the BDNF rs6265 (Val66Met) polymorphism and environment risk factors on psoriasis vulgaris. Mol Biol Rep. 2014;41: 7015–7022. doi:10.1007/s11033-014-3589-4

Danielsen K, Wilsgaard T, Olsen AO, Eggen AE, Olsen K, Cassano PA, et al. Elevated odds of metabolic syndrome in psoriasis: a population-based study of age and sex differences. Br J Dermatol. Wiley/Blackwell (10.1111); 2015;172: 419–427. doi:10.1111/bjd.13288

Dhara S, Dasgupta A, Rout JK, Banerjee U, Dasgupta S, Ghosh A. Clinico-biochemical correlation between psoriasis and insulin resistance. Indian J Clin Biochem. Springer; 2015;30: 99–103. doi:10.1007/s12291-013-0413-1

Owczarczyk-Saczonek AB, Nowicki RJ. Original paper Prevalence of cardiovascular disease risk factors, and metabolic syndrome and its components in patients with psoriasis aged 30 to 49 years. Adv Dermatology Allergol. 2015;4: 290–295. doi:10.5114/pdia.2014.40966

Gisondi P, Barba E, Girolomoni G. Non-alcoholic fatty liver disease fibrosis score in patients with psoriasis. J Eur Acad Dermatology Venereol. Wiley/Blackwell (10.1111); 2016;30: 282–287. doi:10.1111/jdv.13456

Kothiwala SK, Khanna N, Tandon N, Naik N, Sharma VK, Sharma S, et al. Prevalence of metabolic syndrome and cardiovascular changes in patients with chronic plaque psoriasis and their correlation with disease severity: A hospital-based cross-sectional study. Indian J Dermatol Venereol Leprol. Medknow Publications and Media Pvt. Ltd.; 2016;82: 510–8. doi:10.4103/0378-6323.183638

Lonnberg AS, Skov L, Skytthe A, Kyvik KO, Pedersen OB, Thomsen SF. Association of Psoriasis With the Risk for Type 2 Diabetes Mellitus and Obesity. JAMA dermatology. American Medical Association; 2016;152: 1–7. doi:10.1001/jamadermatol.2015.6262

Naito R, Imafuku S. Distinguishing features of body mass index and psoriasis in men and women in Japan: A hospital-based case-control study. J Dermatol. Wiley/Blackwell (10.1111); 2016;43: 1406–1411. doi:10.1111/1346-8138.13439

Praveenkumar U, Ganguly S, Ray L, Nanda SK, Kuruvila S. Prevalence of Metabolic Syndrome in Psoriasis Patients and its Relation to Disease Duration: A Hospital Based Case-Control Study. J Clin Diagn Res. JCDR Research & Publications Private Limited; 2016;10: WC01-5. doi:10.7860/JCDR/2016/17791.7218

Paediatric psoriasis studies

Özden MG, Tekin NS, Gürer MA, Akdemir D, Doğramacı Ç, Utaş S, et al. Environmental Risk Factors in Pediatric Psoriasis: A Multicenter Case-Control Study. Pediatr Dermatol. Wiley/Blackwell (10.1111); 2011;28: 306–312. doi:10.1111/j.1525-1470.2011.01408.x

Goldminz AM, Buzney CD, Kim N, Au S-C, Levine DE, Wang AC, et al. Prevalence of the Metabolic Syndrome in Children with Psoriatic Disease. Pediatr Dermatol. 2013;30: 700–705. doi:10.1111/pde.12218

Torres T, Machado S, Mendonça D, Selores M. Cardiovascular comorbidities in childhood psoriasis. Eur J Dermatology. 2014;24: 229–235. doi:10.1684/EJD.2014.2291

Ergun T, Seckin Gencosmanoglu D, Karakoc-Aydiner E, Salman A, Tekin B, Bulbul-Baskan E, et al. Prevalence of obesity in paediatric psoriasis and its impact on disease severity and progression. Australas J Dermatol. Wiley/Blackwell (10.1111); 2017;58: e182–e187. doi:10.1111/ajd.12491

Supporting Figures

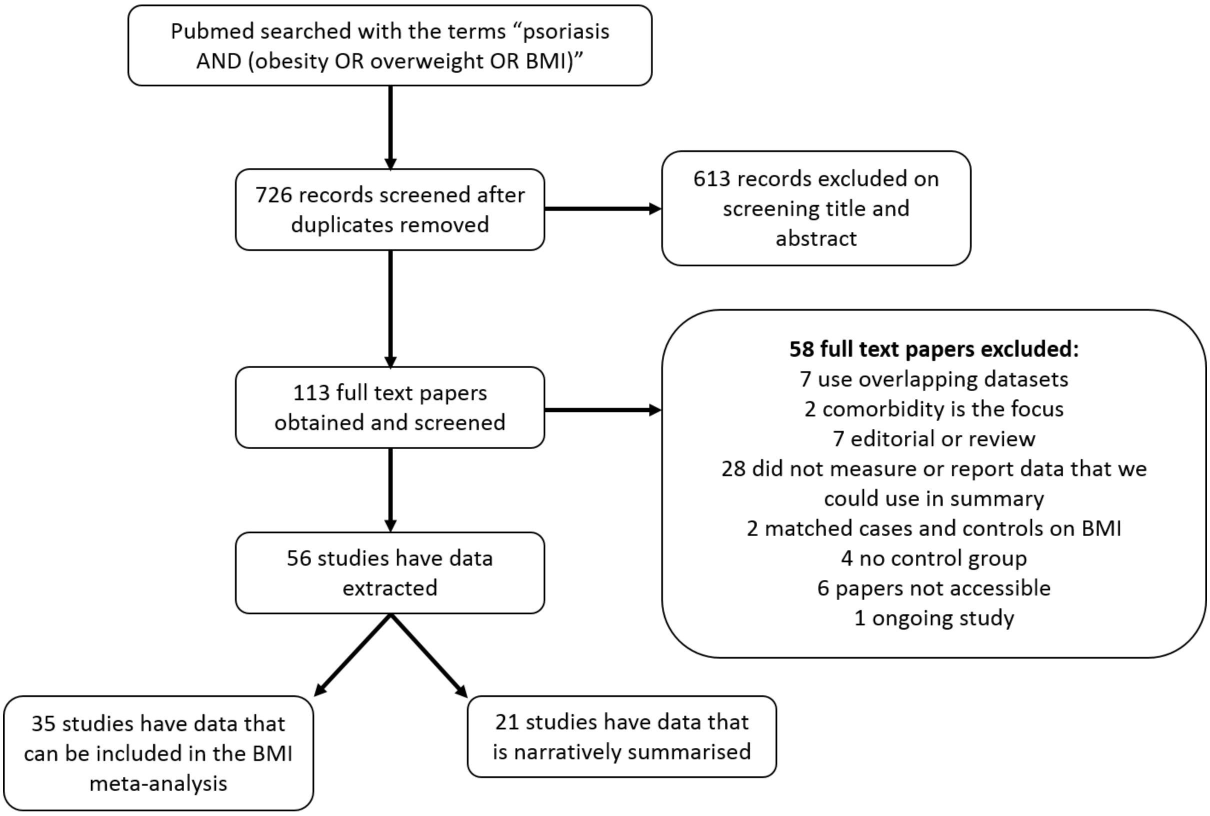

Fig A. Flow diagram for literature review

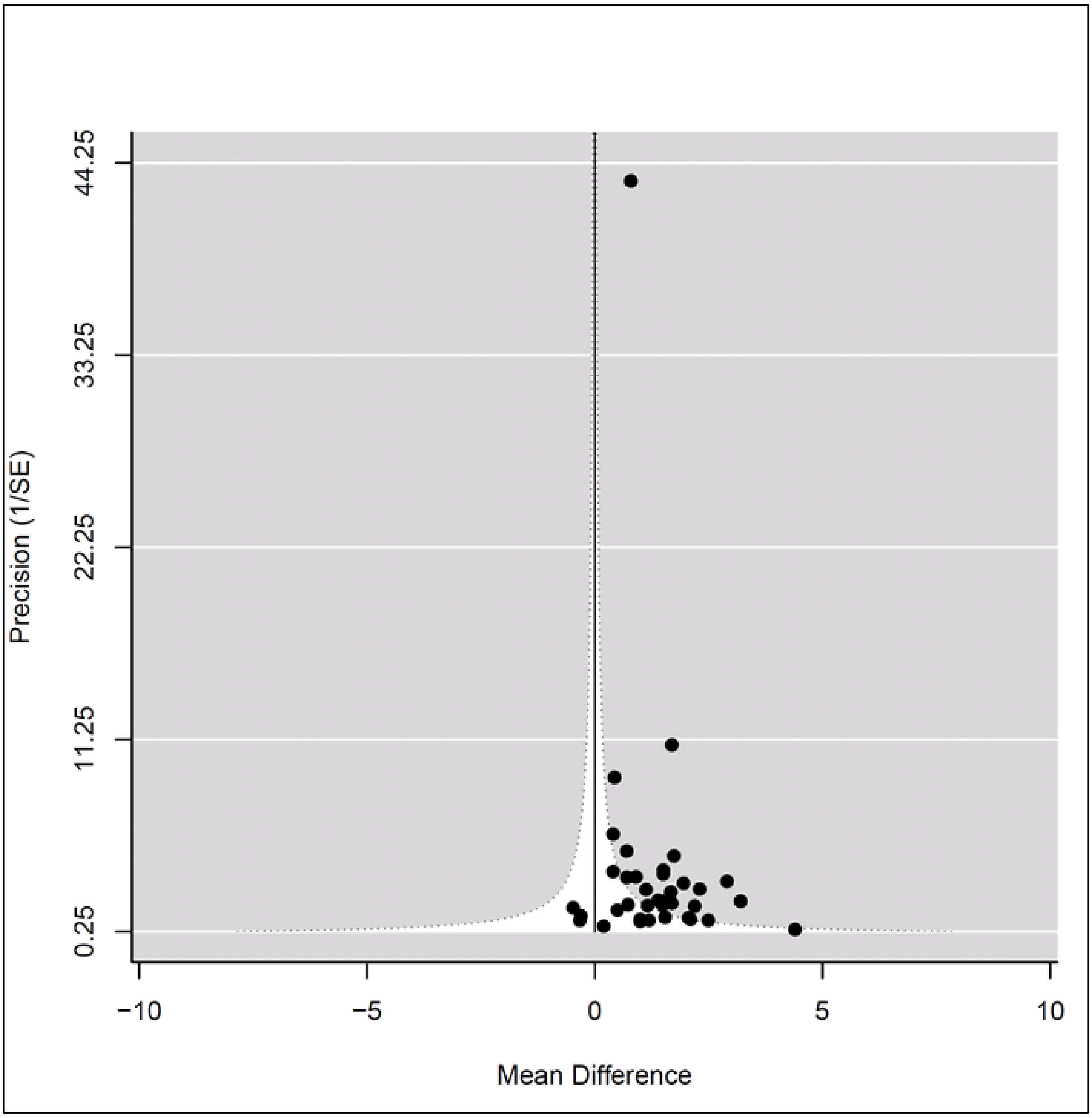

Fig B. Bias assessment plot to assess publication bias in meta-analysis of published observational associations between BMI and psoriasis. Bias=0.23, 95% CI -0.64, 1.10; *P*=0.60
SE=standard error

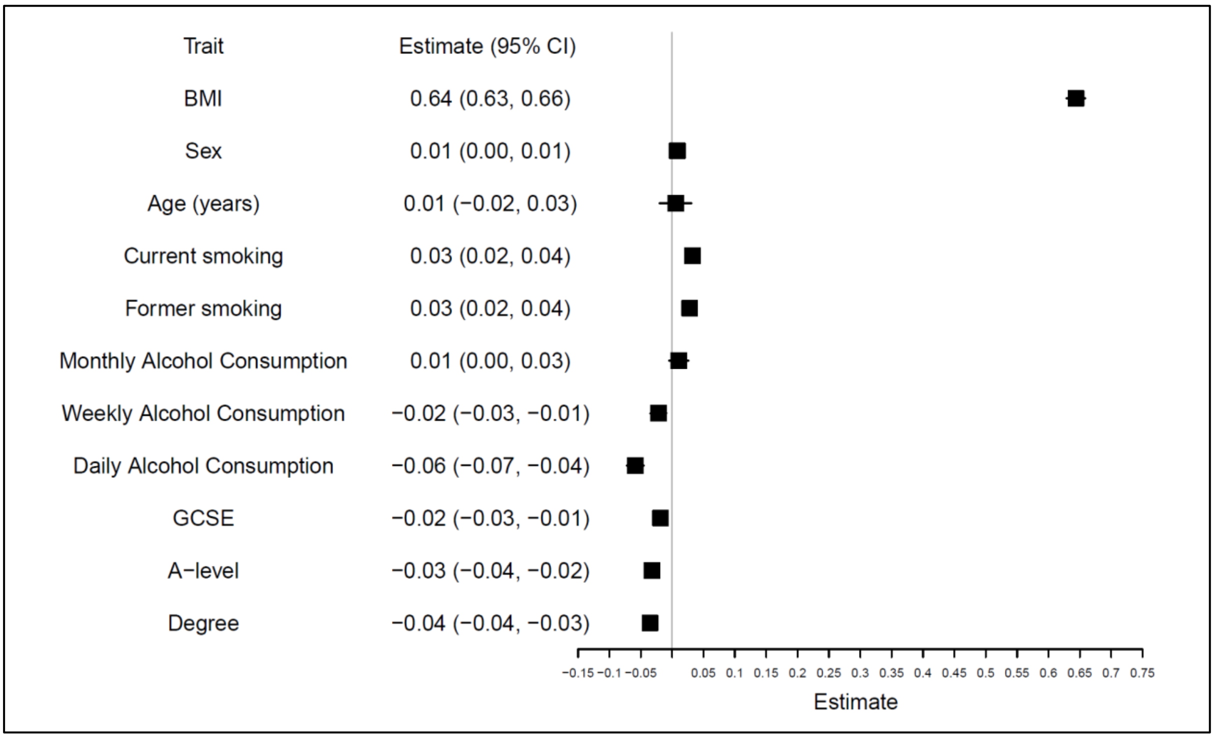

Fig C. Association of BMI GRS with BMI (kg/m^2) and potential confounders in UK Biobank. Estimates are given per 1 standard deviation increase in BMI GRS. A-level, Advanced level studies; CI, confidence interval; GCSE, General Certificate of Secondary Education; Monthly Alcohol Consumption was defined as frequency of "one to three times a month".

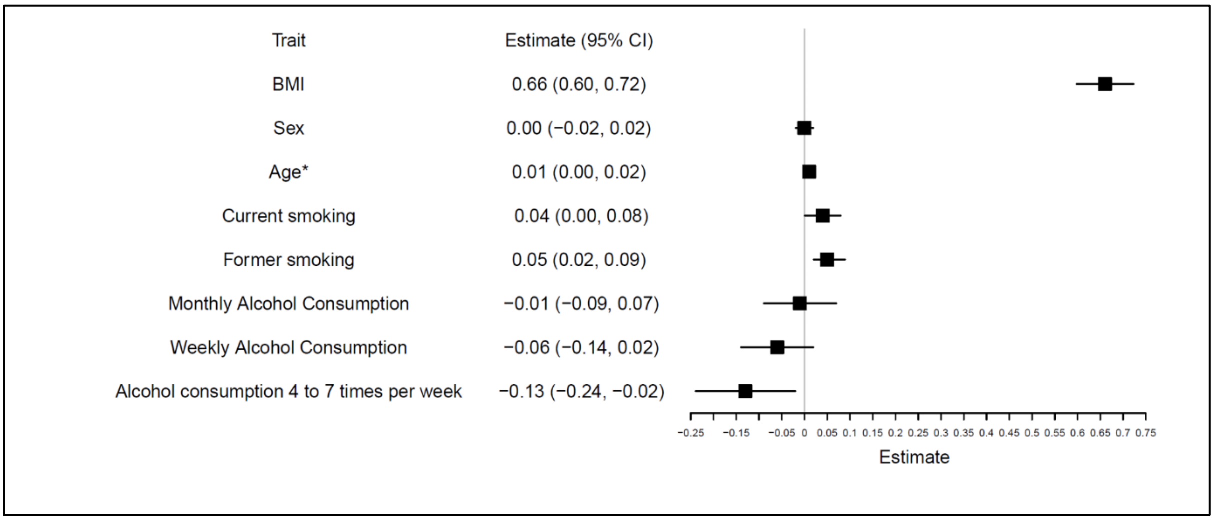

Fig D. Association of BMI GRS with BMI (kg/m^2) and potential confounders in HUNT. Estimates are given per 1 standard deviation increase in BMI GRS. BMI, body mass index; CI, confidence interval. *age given per 10 year intervals

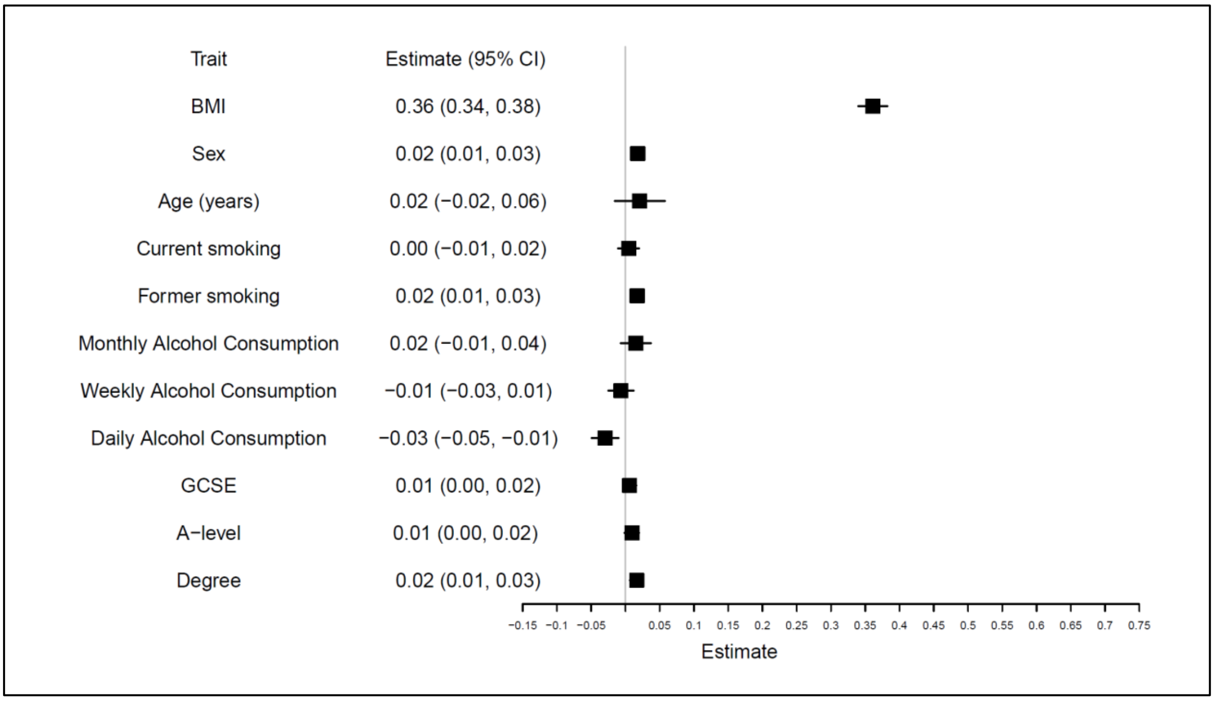

Fig E. Association of FTO SNP (rs1558902) with BMI (kg/m^2) and potential confounders in UK Biobank. Estimates are given per 1 copy increase in effect allele (*A*).
A-level, Advanced level studies; CI, confidence interval; GCSE, General Certificate of Secondary Education; Monthly Alcohol Consumption, defined as frequency of "one to three times a month".

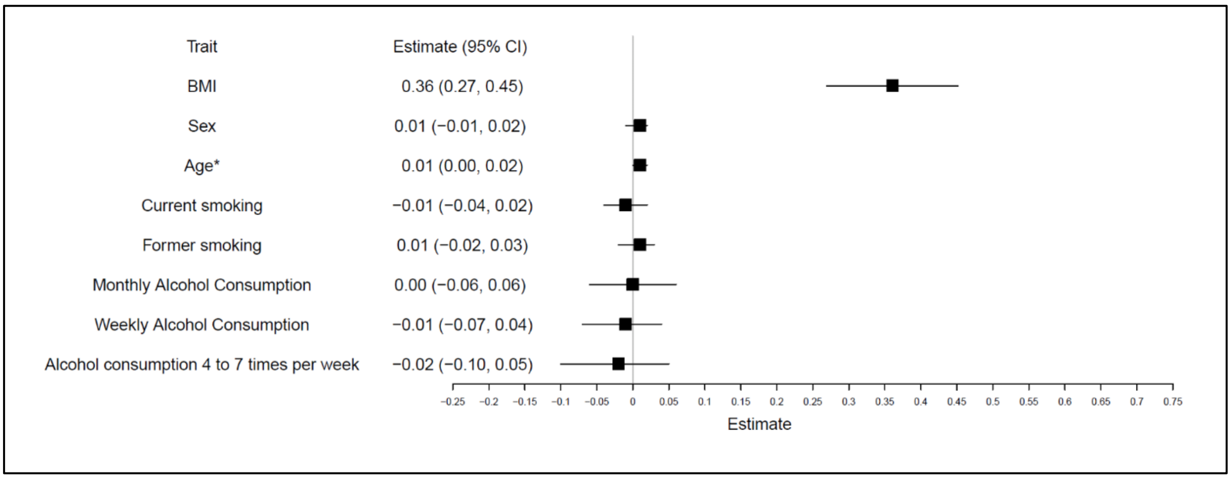

Fig F. Association of FTO SNP (rs1558902) with BMI (kg/m^2) and potential confounders in HUNT. Estimates are given per 1 copy increase in effect allele (*A*). *age given per 10 year intervals

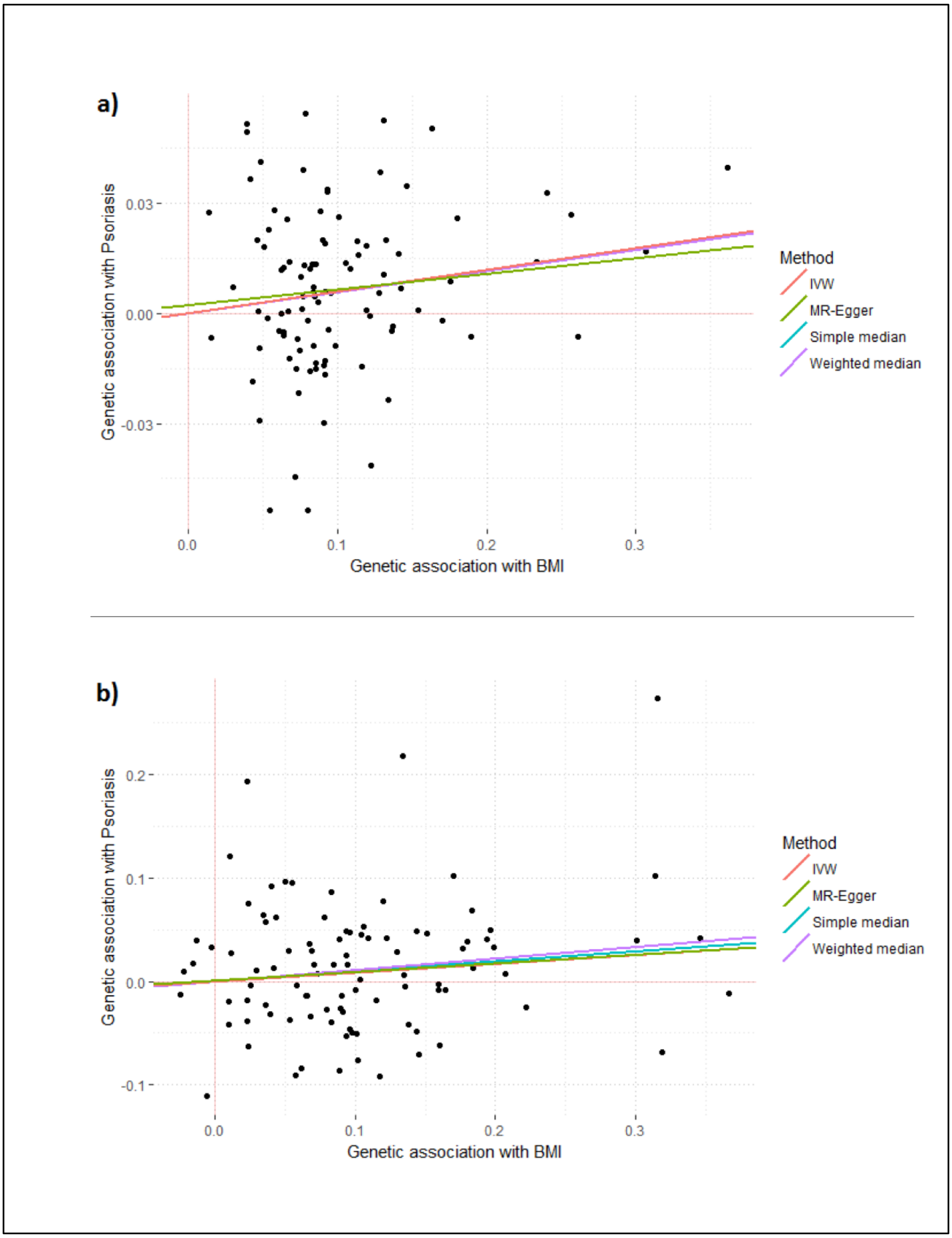

Fig G. Associations of BMI SNPs with BMI and psoriasis within a) UK Biobank b) HUNT. IVW, MR-Egger, weighted median and simple median estimates are indicated by the red, green, purple and blue lines respectively.

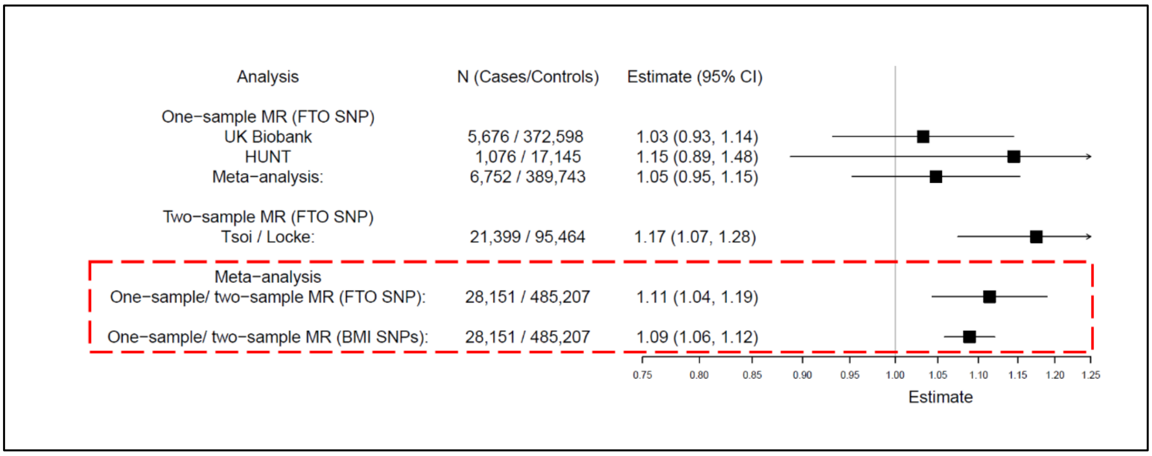

Fig H. Effect of FTO variant, rs1558902 upon psoriasis. Results from MR analysis using rs1558902 as an instrumental variable. These are compared with final estimates obtained when using all BMI SNPs as a genetic instrument. One-sample MR was performed with individual-level data from UK Biobank and HUNT. Two-sample MR was performed with published GWAS summary-level data for BMI and psoriasis. Estimates are given per 1 unit increase in BMI (kg/m^2).

a)

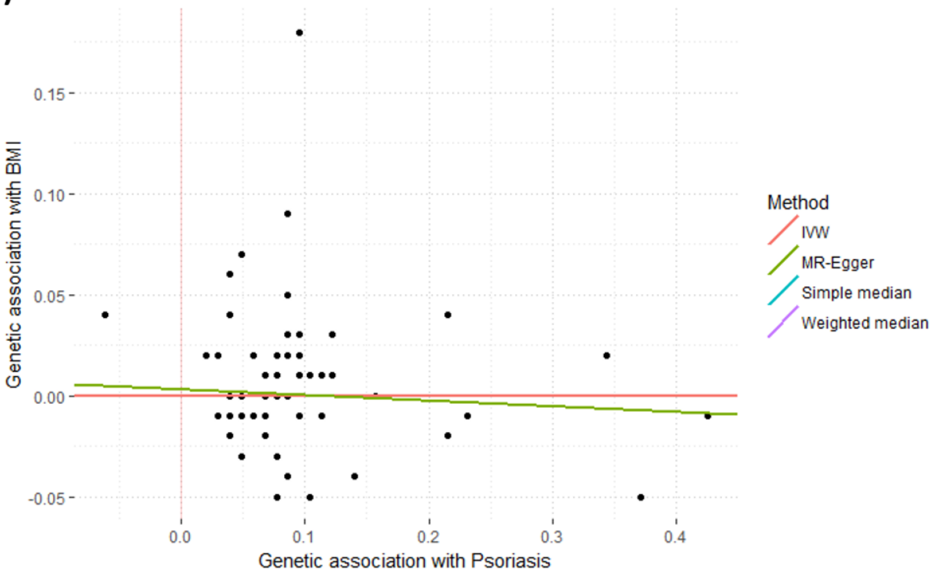

b)

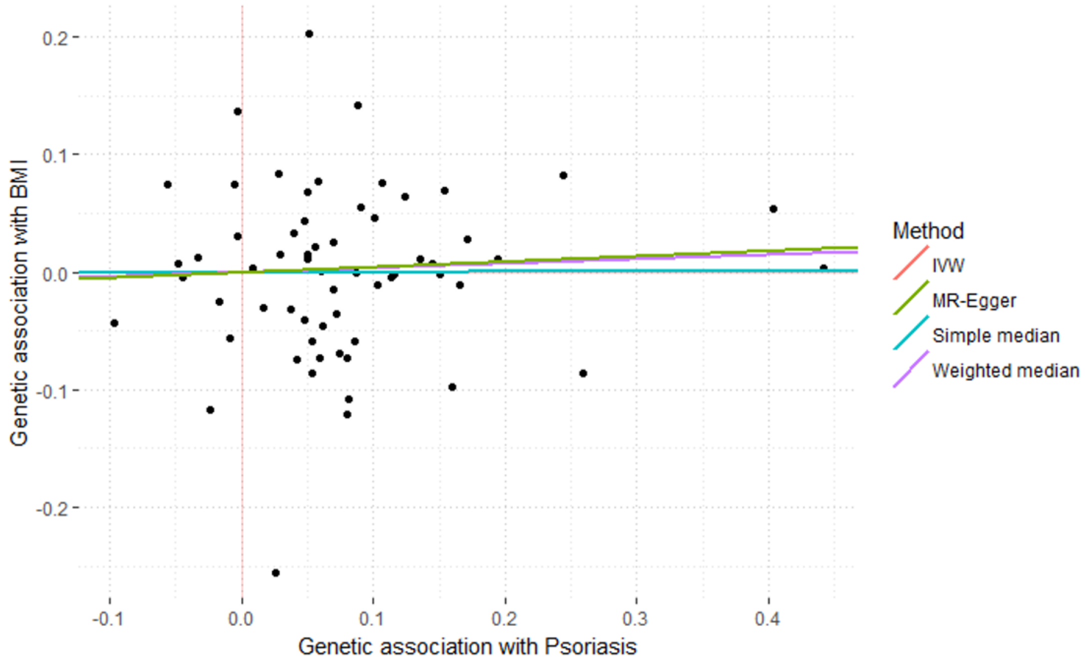

Fig I. Association of psoriasis SNPs with psoriasis and BMI within (a) UK Biobank and (b) HUNT. IVW, MR-Egger, weighted median and simple median estimates are indicated by the red, green, purple and blue lines respectively.

Supporting Tables

Table A. International Classification of Diseases (ICD)-10 codes used to obtain disease information for psoriasis from the UK Biobank resource

Phenotype	ICD10 codes
Psoriasis vulgaris	L40-L400
Generalised pustular psoriasis	L40-L401
Acrodermatitis continua	L40-L402
Pustulosis palmaris et plantaris	L40-L403
Guttate psoriasis	L40-L404
Arthropathic psoriasis	L40-L405
Other psoriasis	L40-L408
Psoriasis, unspecified	L40-409

Table B. Association of BMI genetic instruments with BMI in UK Biobank and HUNT

BMI variants reported by Locke et al (2015)

				BMI[a] (UK Biobank)		BMI[a] (HUNT)		BMI[b] (Locke 2015)	
SNP	CHR	Position (bp)	Effect allele	Beta (95% CI)	*P* value	Beta (95% CI)	*P* value	Beta (95% CI)	*P* value
rs977747	1	47684677	T	0.08 (0.06,0.10)	1.24E-13	0.03 (-0.06,0.12)	0.53	0.02 (0.01,0.02)	8.65E-08
rs657452	1	49589847	A	0.08 (0.06,0.10)	5.38E-12	0.09 (0.00,0.19)	0.05	0.02 (0.02,0.03)	5.48E-13
rs11583200	1	50559820	C	0.07 (0.05,0.09)	1.48E-09	0.08 (-0.01,0.18)	0.08	0.02 (0.01,0.02)	1.48E-08
rs3101336	1	72751185	C	0.11 (0.08,0.13)	1.68E-21	0.18 (0.09,0.27)	8.96E-05	0.03 (0.03,0.04)	2.66E-26
rs12566985	1	75002193	G	0.08 (0.06,0.11)	1.36E-14	0.09 (0.00,0.18)	0.05	0.02 (0.02,0.03)	3.28E-15
rs12401738	1	78446761	A	0.08 (0.06,0.10)	1.17E-12	0.08 (-0.01,0.18)	0.07	0.02 (0.02,0.03)	1.15E-10
rs11165643	1	96924097	T	0.09 (0.06,0.11)	8.99E-15	0.05 (-0.04,0.14)	0.26	0.02 (0.02,0.03)	2.07E-12
rs17024393	1	110154688	C	0.31 (0.24,0.37)	6.26E-19	0.31 (0.03,0.59)	0.03	0.07 (0.05,0.08)	7.03E-14
rs543874	1	177889480	G	0.24 (0.21,0.27)	3.36E-71	0.37 (0.26,0.47)	1.03E-11	0.05 (0.04,0.06)	2.62E-35
rs2820292	1	201784287	C	0.09 (0.07,0.11)	5.58E-17	0.12 (0.03,0.21)	0.01	0.02 (0.01,0.03)	1.83E-10
rs13021737	2	632348	G	0.26 (0.23,0.29)	3.95E-74	0.19 (0.07,0.31)	1.72E-03	0.06 (0.05,0.07)	1.11E-50
rs10182181	2	25150296	G	0.17 (0.15,0.19)	2.39E-55	0.21 (0.12,0.30)	6.30E-06	0.03 (0.03,0.04)	8.78E-24
rs11126666	2	26928811	A	0.01 (-0.01,0.04)	2.29E-01	0.01 (-0.09,0.11)	0.82	0.02 (0.02,0.03)	1.33E-09
rs1016287	2	59305625	T	0.09 (0.07,0.12)	5.07E-15	0.15 (0.05,0.25)	2.78E-03	0.02 (0.02,0.03)	2.25E-11
rs11688816	2	63053048	G	0.05 (0.03,0.07)	2.24E-05	-0.02 (-0.12,0.07)	0.60	0.02 (0.01,0.02)	1.89E-08
rs2121279	2	143043285	T	0.05 (0.02,0.08)	2.18E-03	-0.01 (-0.14,0.12)	0.93	0.02 (0.02,0.03)	2.31E-08
rs1460676	2	164567689	C	0.06 (0.03,0.09)	2.24E-05	0.06 (-0.07,0.18)	0.36	0.02 (0.01,0.03)	8.98E-07
rs1528435	2	181550962	T	0.08 (0.05,0.10)	8.76E-12	0.07 (-0.02,0.17)	0.12	0.02 (0.01,0.02)	1.2E-08
rs17203016	2	208255518	G	0.06 (0.04,0.09)	2.67E-06	0.04 (-0.07,0.15)	0.47	0.02 (0.01,0.03)	8.15E-08
rs7599312	2	213413231	G	0.08 (0.05,0.10)	7.65E-10	0.11 (0.01,0.21)	0.03	0.02 (0.02,0.03)	1.17E-10
rs492400	2	219349752	C	0.05 (0.03,0.08)	9.08E-07	0.07 (-0.02,-0.16)	0.15	0.02 (0.01,0.02)	4.17E-07
rs2176040	2	227092802	A	0.01 (-0.01,0.04)	0.23	-0.02 (-0.11,0.07)	0.64	0.01 (0.01,0.02)	6.06E-06

rs6804842	3	25106437	G	0.06 (0.04,0.09)	7.57E-09	0.10 (0.01,0.19)	0.03	0.02 (0.01,0.02)	2.48E-09
rs2365389	3	61236462	C	0.07 (0.05,0.09)	8.70E-11	0.04 (-0.05,0.13)	0.37	0.02 (0.01,0.03)	1.63E-10
rs3849570	3	81792112	A	0.06 (0.04,0.08)	4.03E-07	0.02 (-0.07,0.12)	0.63	0.02 (0.01,0.02)	2.6E-08
rs13078960	3	85807590	G	0.09 (0.06,0.12)	2.06E-11	0.09 (-0.02,0.20)	0.12	0.03 (0.02,0.04)	1.74E-14
rs16851483	3	141275436	T	0.18 (0.14,0.22)	1.45E-16	0.01 (-0.18,0.20)	0.91	0.05 (0.03,0.06)	3.55E-10
rs1516725	3	185824004	C	0.16 (0.13,0.19)	4.80E-25	0.13 (-0.01,0.27)	0.06	0.04 (0.04,0.05)	1.89E-22
rs10938397	4	45182527	G	0.15 (0.13,0.17)	7.36E-41	0.18 (0.08,0.27)	1.59E-04	0.04 (0.03,0.05)	3.21E-38
rs17001654	4	77129568	G	0.08 (0.05,0.11)	3.14E-07	0.14 (0.02,0.27)	0.03	0.03 (0.02,0.04)	7.76E-09
rs13107325	4	103188709	T	0.23 (0.19,0.27)	7.60E-30	0.13 (-0.07,0.33)	0.21	0.05 (0.03,0.06)	1.83E-12
rs11727676	4	145659064	T	0.04 (0.00,0.07)	3.42E-02	0.12 (-0.04,0.28)	0.13	0.04 (0.02,0.05)	2.55E-08
rs2112347	5	75015242	T	0.14 (0.11,0.16)	1.03E-33	0.04 (-0.05,0.14)	0.37	0.03 (0.02,0.03)	6.19E-17
rs7715256	5	153537893	G	0.08 (0.05,0.10)	6.96E-12	0.08 (-0.01,0.17)	0.09	0.02 (0.01,0.02)	1.7E-07
rs205262	6	34563164	G	0.14 (0.12,0.16)	2.69E-30	0.20 (0.09,0.30)	1.6E-04	0.02 (0.01,0.03)	1.75E-10
rs2033529	6	40348653	G	0.10 (0.07,0.12)	2.60E-16	0.02 (-0.08,0.12)	0.64	0.02 (0.01,0.02)	1.39E-08
rs2207139	6	50845490	G	0.19 (0.16,0.22)	2.57E-39	0.16 (0.04,0.28)	7.98E-03	0.04 (0.04,0.05)	4.13E-29
rs9400239	6	108977663	C	0.08 (0.06,0.11)	6.69E-12	0.08 (-0.02,0.18)	0.13	0.02 (0.01,0.02)	1.61E-08
rs9374842	6	120185665	T	0.06 (0.04,0.09)	2.15E-06	0.01 (-0.09,0.11)	0.85	0.02 (0.02,0.03)	2.67E-08
rs13201877	6	137675541	G	0.04 (0.01,0.07)	1.58E-02	0.00(-0.13,0.12)	0.96	0.02 (0.01,0.03)	2.35E-07
rs13191362	6	163033350	A	0.10 (0.06,0.13)	8.09E-09	0.16 (0.03,0.30)	0.01	0.03 (0.02,0.04)	7.34E-09
rs1167827	7	75163169	G	0.10 (0.08,0.12)	2.99E-20	0.07 (-0.02,0.16)	0.13	0.02 (0.01,0.03)	6.33E-10
rs2245368	7	76608143	C	0.12 (0.09,0.15)	3.76E-17	0.16 (0.04,0.28)	8.69E-03	0.03 (0.02,0.04)	3.19E-08
rs9641123	7	93197732	C	0.04 (0.02,0.06)	1.83E-04	0.09 (0.00,0.18)	0.06	0.03 (0.02,0.04)	2.08E-10
rs6465468	7	95169514	T	0.03 (0.01,0.05)	1.09E-02	0.06 (-0.04,0.16)	0.21	0.02 (0.01,0.02)	2.32E-06
rs17405819	8	76806584	T	0.09 (0.07,0.12)	2.01E-15	0.11 (0.02,0.21)	0.02	0.02 (0.02,0.03)	2.07E-11
rs16907751	8	81375457	C	0.09 (0.06,0.13)	7.25E-07	0.02 (-0.13,0.17)	0.77	0.04 (0.02,0.05)	1.26E-07
rs2033732	8	85079709	C	0.05 (0.02,0.07)	1.01E-04	0.02 (-0.08,0.13)	0.66	0.02 (0.01,0.03)	4.89E-08
rs4740619	9	15634326	T	0.09 (0.07,0.11)	1.24E-15	0.13 (0.04,0.22)	3.45E-03	0.02 (0.01,0.02)	4.56E-09

rs10968576	9	28414339	G	0.12 (0.10,0.14)	5.79E-26	0.14 (0.04,0.23)	0.01	0.02 (0.02,0.03)	6.61E-14
rs6477694	9	111932342	C	0.06 (0.04,0.08)	4.80E-08	0.04 (-0.05,0.13)	0.40	0.02 (0.01,0.02)	2.67E-08
rs1928295	9	120378483	T	0.06 (0.04,0.08)	1.19E-08	0.10 (0.01,0.19)	0.03	0.02 (0.01,0.02)	7.91E-10
rs10733682	9	129460914	A	0.07 (0.04,0.09)	4.20E-09	0.07 (-0.02,0.15)	0.16	0.02 (0.01,0.02)	1.83E-08
rs7899106	10	87410904	G	0.13 (0.09,0.18)	8.60E-08	0.32 (0.09,0.54)	5.45E-03	0.04 (0.03,0.05)	2.96E-08
rs17094222	10	102395440	C	0.07 (0.04,0.09)	4.41E-07	0.09 (-0.02,0.21)	0.11	0.02 (0.02,0.03)	5.94E-11
rs11191560	10	104869038	C	0.12 (0.08,0.16)	3.49E-09	-0.01 (-0.18,0.15)	0.87	0.03 (0.02,0.04)	8.45E-09
rs7903146	10	114758349	C	0.08 (0.06,0.11)	2.38E-12	0.15 (0.04,0.25)	5.79E-03	0.02 (0.02,0.03)	1.1E-11
rs4256980	11	8673939	G	0.09 (0.06,0.11)	8.36E-14	0.10 (0.00,0.19)	0.04	0.02 (0.02,0.03)	2.9E-11
rs11030104	11	27684517	A	0.18 (0.15,0.20)	7.14E-39	0.09 (-0.03,0.20)	0.13	0.04 (0.03,0.05)	5.56E-28
rs2176598	11	43864278	T	0.09 (0.06,0.11)	2.10E-12	0.10 (-0.01,0.20)	0.07	0.02 (0.01,0.03)	2.97E-08
rs3817334	11	47650993	T	0.11 (0.09,0.14)	6.01E-25	0.10 (0.00,0.19)	0.04	0.03 (0.02,0.03)	5.15E-17
rs12286929	11	115022404	G	0.08 (0.06,0.10)	1.97E-14	0.10 (0.01,0.19)	0.03	0.02 (0.02,0.03)	1.31E-12
rs7138803	12	50247468	A	0.13 (0.11,0.15)	4.35E-32	0.14 (0.05,0.23)	3.21E-03	0.03 (0.03,0.04)	8.15E-24
rs11057405	12	122781897	G	0.13 (0.10,0.17)	1.76E-13	0.18 (0.03,0.33)	0.02	0.03 (0.02,0.04)	2.02E-08
rs9581854*	13	28017782	T	0.05 (0.02,0.07)	9.85E-04	0.07 (-0.04,0.18)	0.24	0.03 (0.02,0.04)	2.29E-10
rs12429545	13	54102206	A	0.14 (0.10,0.17)	1.44E-16	0.16 (0.03,0.29)	0.02	0.03 (0.02,0.04)	1.09E-12
rs9540493	13	66205704	A	0.07 (0.05,0.09)	6.31E-10	0.02 (-0.07,0.11)	0.61	0.02 (0.01,0.03)	4.97E-08
rs1441264	13	79580919	A	0.09 (0.07,0.11)	2.73E-15	0.05 (-0.04,0.14)	0.29	0.02 (0.01,0.02)	6.04E-08
rs10132280	14	25928179	C	0.11 (0.09,0.14)	1.41E-21	0.10 (0.01,0.20)	0.04	0.02 (0.02,0.03)	1.14E-11
rs12885454	14	29736838	C	0.07 (0.05,0.10)	5.74E-11	0.05 (-0.04,0.15)	0.25	0.02 (0.02,0.03)	1.94E-10
rs11847697	14	30515112	T	0.12 (0.06,0.17)	1.11E-05	0.32 (0.07,0.57)	0.01	0.05 (0.03,0.06)	3.99E-09
rs7141420	14	79899454	T	0.09 (0.07,0.11)	1.69E-16	0.10 (0.01,0.19)	0.04	0.02 (0.02,0.03)	1.23E-14
rs3736485	15	51748610	A	0.07 (0.05,0.09)	3.49E-11	0.01 (-0.08,0.10)	0.83	0.02 (0.01,0.02)	7.41E-09
rs16951275	15	68077168	T	0.13 (0.11,0.16)	5.11E-24	0.17 (0.06,0.28)	2.36E-03	0.03 (0.02,0.04)	1.91E-17
rs7164727	15	73093991	T	0.08 (0.06,0.10)	1.86E-11	0.07 (-0.03,0.17)	0.16	0.02 (0.01,0.02)	6.83E-08
rs758747	16	3627358	T	0.05 (0.03,0.08)	1.83E-05	0.11 (0.00,0.21)	0.05	0.02 (0.02,0.03)	7.47E-10

rs12446632	16	19935389	G	0.14 (0.11,0.17)	5.18E-20	0.22 (0.09,0.36)	1.15E-03	0.04 (0.03,0.05)	1.48E-18
rs2650492	16	28333411	A	0.09 (0.07,0.12)	2.54E-14	0.18 (0.09,0.28)	2.22E-04	0.02 (0.01,0.03)	1.92E-09
rs3888190	16	28889486	A	0.13 (0.11,0.15)	8.47E-31	0.20 (0.11,0.29)	1.94E-05	0.03 (0.03,0.04)	3.14E-23
rs4787491	16	30015337	G	0.08 (0.06,0.11)	6.15E-15	0.14 (0.05,0.23)	1.69E-03	0.02 (0.01,0.03)	2.7E-08
rs9925964	16	31129895	A	0.12 (0.10,0.14)	6.83E-26	0.04 (-0.06,0.13)	0.46	0.02 (0.01,0.02)	8.11E-10
rs2080454	16	49062590	C	0.05 (0.03,0.07)	2.31E-05	0.09 (0.00,0.18)	0.04	0.02 (0.01,0.02)	6.55E-08
rs1558902	16	53803574	A	0.36 (0.34,0.38)	5.99E-234	0.35 (0.26,0.44)	7.66E-14	0.08 (0.08,0.09)	7.5E-153
rs9914578	17	2005136	G	0.04 (0.02,0.07)	1.33E-03	0.09 (-0.01,0.20)	0.09	0.02 (0.01,0.03)	8.99E-08
rs1000940	17	5283252	G	0.07 (0.05,0.10)	7.48E-10	0.03 (-0.07,0.12)	0.61	0.02 (0.01,0.02)	1.28E-08
rs12940622	17	78615571	G	0.08 (0.06,0.11)	1.33E-14	0.09 (0.00,0.18)	0.05	0.02 (0.01,0.02)	2.49E-09
rs1808579	18	21104888	C	0.11 (0.09,0.13)	1.91E-23	0.12 (0.03,0.21)	8.84E-03	0.02 (0.01,0.02)	4.17E-08
rs7239883	18	40147671	G	0.05 (0.03,0.07)	1.93E-05	0.03 (-0.06,0.13)	0.46	0.02 (0.01,0.02)	1.63E-07
rs7243357	18	56883319	T	0.09 (0.06,0.11)	2.03E-09	0.05 (-0.07,0.18)	0.41	0.02 (0.01,0.03)	3.86E-08
rs6567160	18	57829135	C	0.26 (0.23,0.28)	3.29E-89	0.30 (0.20,0.40)	8.32E-09	0.06 (0.05,0.06)	3.93E-53
rs17724992	19	18454825	A	0.07 (0.05,0.10)	1.77E-09	-0.02 (-0.12,0.09)	0.76	0.02 (0.01,0.03)	3.42E-08
rs29941	19	34309532	G	0.08 (0.06,0.10)	1.32E-11	0.12 (0.02,0.21)	0.02	0.02 (0.01,0.02)	2.41E-08
rs2075650	19	45395619	A	0.09 (0.06,0.12)	1.35E-09	0.08 (-0.04,0.21)	0.19	0.03 (0.02,0.04)	1.25E-08
rs2287019	19	46202172	C	0.15 (0.13,0.18)	1.75E-27	0.11 (0.00,0.22)	0.05	0.04 (0.03,0.04)	4.59E-18
rs3810291	19	47569003	A	0.13 (0.11,0.15)	8.19E-29	0.07 (-0.03,0.16)	0.16	0.03 (0.02,0.04)	4.81E-15
rs6091540	20	51087862	C	0.09 (0.07,0.11)	2.95E-14	0.04 (-0.06,0.13)	0.47	0.02 (0.01,0.02)	2.32E-06
rs2836754	21	40291740	C	0.05 (0.03,0.08)	1.29E-06	0.06 (-0.04,0.15)	0.23	0.02 (0.01,0.02)	4.16E-07

[a]Estimate given for raw BMI (kg/m^2); [b]Estimate given for inverse-transformed BMI; Bp, base position; CHR, chromosome; CI, confidence interval; *proxy SNP for reported SNP (see S4 Table)

Table C. Association of BMI genetic instruments with psoriasis in UK Biobank and HUNT

BMI variants reported by Locke et al (2015)

				Psoriasis (UK Biobank)		Psoriasis (HUNT)		Psoriasis (Tsoi 2017)	
**SNP**	**CHR**	**Position (bp)**	**Effect allele**	**OR (95% CI)**	***P* value**	**OR (95% CI)**	***P* value**	**OR (95% CI)**	***P* value**
rs977747	1	47684677	T	0.99 (0.96,1.03)	0.75	1.01 (0.92,1.11)	0.81	0.99 (0.95,1.02)	0.42
rs657452	1	49589847	A	1.00 (0.97,1.04)	0.82	1.05 (0.96,1.15)	0.29	1.02 (0.98,1.07)	0.26
rs11583200	1	50559820	C	0.99 (0.95,1.02)	0.46	1.09 (1.00,1.20)	0.05	0.97 (0.93,1.01)	0.12
rs3101336	1	72751185	C	1.00 (0.96,1.04)	0.96	1.07 (0.98,1.17)	0.13	1.00 (0.96,1.05)	0.86
rs12566985	1	75002193	G	1.06 (1.02,1.10)	4.11E-03	0.97 (0.89,1.06)	0.51	1.01 (0.97,1.05)	0.58
rs12401738	1	78446761	A	1.02 (0.98,1.06)	0.40	1.02 (0.93,1.11)	0.73	1.07 (1.03,1.11)	1.64E-04
rs11165643	1	96924097	T	0.97 (0.94,1.01)	0.13	1.03 (0.94,1.13)	0.51	1.01 (0.97,1.05)	0.58
rs17024393	1	110154688	C	0.95 (0.84,1.07)	0.38	1.11 (0.85,1.43)	0.44	0.89 (0.79,1.00)	0.06
rs543874	1	177889480	G	0.99 (0.95,1.04)	0.71	0.99 (0.90,1.09)	0.82	1.04 (0.99,1.09)	0.11
rs2820292	1	201784287	C	1.03 (0.99,1.07)	0.15	1.01 (0.92,1.11)	0.79	1.01 (0.97,1.05)	0.73
rs13021737	2	632348	G	1.04 (0.99,1.09)	0.17	1.04 (0.92,1.17)	0.50	0.98 (0.94,1.02)	0.38
rs10182181	2	25150296	G	1.03 (1.00,1.07)	0.08	1.01 (0.92,1.09)	0.88	0.95 (0.92,0.98)	1.50E-03
rs11126666	2	26928811	A	1.00 (0.96,1.04)	0.96	1.03 (0.93,1.13)	0.58	1.01 (0.97,1.05)	0.70
rs1016287	2	59305625	T	1.04 (1.00,1.08)	0.06	1.05 (0.95,1.15)	0.33	1.01 (0.97,1.06)	0.57
rs11688816	2	63053048	G	1.02 (0.98,1.06)	0.30	0.99 (0.90,1.07)	0.78	0.97 (0.93,1.01)	0.09
rs2121279	2	143043285	T	0.98 (0.93,1.04)	0.59	0.89 (0.79,1.02)	0.09	1.06 (1.00,1.12)	0.05
rs1460676	2	164567689	C	0.98 (0.93,1.03)	0.48	1.00 (0.89,1.13)	0.96	1.00 (0.94,1.05)	0.86

rs1528435	2	181550962	T	0.99 (0.95,1.03)	0.63	1.01 (0.92,1.11)	0.87	1.01 (0.97,1.05)	0.56
rs17203016	2	208255518	G	1.00 (0.95,1.05)	0.95	1.10 (0.99,1.22)	0.08	1.01 (0.96,1.06)	0.61
rs7599312	2	213413231	G	1.00 (0.96,1.05)	0.83	1.04 (0.95,1.15)	0.39	1.00 (0.96,1.05)	0.87
rs492400	2	219349752	C	1.02 (0.98,1.06)	0.29	1.04 (0.95,1.13)	0.42	0.99 (0.96,1.03)	0.77
rs2176040	2	227092802	A	0.99 (0.96,1.03)	0.73	1.01 (0.92,1.11)	0.83	1.00 (0.96,1.03)	0.80
rs6804842	3	25106437	G	0.99 (0.95,1.03)	0.52	1.00 (0.91,1.09)	0.97	1.04 (1.00,1.08)	0.05
rs2365389	3	61236462	C	0.99 (0.96,1.03)	0.74	1.01 (0.92,1.11)	0.78	1.01 (0.97,1.05)	0.65
rs3849570	3	81792112	A	1.02 (0.98,1.06)	0.31	0.96 (0.88,1.05)	0.41	0.99 (0.95,1.03)	0.56
rs13078960	3	85807590	G	0.99 (0.94,1.03)	0.53	0.92 (0.82,1.03)	0.13	0.97 (0.93,1.01)	0.19
rs16851483	3	141275436	T	1.04 (0.97,1.12)	0.29	1.13 (0.95,1.35)	0.18	1.09 (1.01,1.18)	0.03
rs1516725	3	185824004	C	1.00 (0.94,1.05)	0.86	1.24 (1.07,1.43)	3.47E-03	0.97 (0.92,1.02)	0.19
rs10938397	4	45182527	G	0.99 (0.95,1.02)	0.48	1.03 (0.94,1.13)	0.48	0.98 (0.95,1.01)	0.25
rs17001654	4	77129568	G	1.03 (0.97,1.08)	0.31	1.05 (0.93,1.19)	0.43	0.99 (0.94,1.04)	0.66
rs13107325	4	103188709	T	1.03 (0.97,1.11)	0.34	1.03 (0.85,1.25)	0.77	1.04 (0.97,1.13)	0.25
rs11727676	4	145659064	T	0.96 (0.90,1.02)	0.16	1.04 (0.90,1.22)	0.60	0.98 (0.91,1.06)	0.61
rs2112347	5	75015242	T	1.02 (0.98,1.06)	0.31	1.06 (0.97,1.16)	0.19	1.04 (1.00,1.08)	0.05
rs7715256	5	153537893	G	0.99 (0.95,1.02)	0.43	0.97 (0.90,1.06)	0.55	0.97 (0.94,1.01)	0.19
rs205262	6	34563164	G	1.01 (0.97,1.06)	0.51	1.05 (0.95,1.16)	0.32	0.99 (0.95,1.03)	0.51
rs2033529	6	40348653	G	1.01 (0.97,1.05)	0.61	1.08 (0.98,1.19)	0.12	1.01 (0.97,1.05)	0.70
rs2207139	6	50845490	G	1.01 (0.97,1.06)	0.59	0.94 (0.84,1.05)	0.30	0.98 (0.93,1.03)	0.38
rs9400239	6	108977663	C	1.01 (0.97,1.06)	0.53	1.06 (0.96,1.17)	0.22	0.97 (0.93,1.02)	0.21

rs9374842	6	120185665	T	1.00 (0.96,1.05)	0.90	0.98 (0.89,1.08)	0.70	1.00 (0.96,1.05)	0.97
rs13201877	6	137675541	G	0.98 (0.93,1.03)	0.44	1.03 (0.92,1.16)	0.58	1.01 (0.96,1.07)	0.67
rs13191362	6	163033350	A	1.01 (0.95,1.06)	0.84	0.99 (0.88,1.13)	0.90	1.02 (0.97,1.09)	0.43
rs1167827	7	75163169	G	1.00 (0.96,1.03)	0.85	1.03 (0.94,1.13)	0.50	1.00 (0.97,1.03)	0.98
rs2245368	7	76608143	C	1.03 (0.98,1.08)	0.29	1.00 (0.89,1.12)	0.96	1.00 (0.92,1.09)	0.96
rs9641123	7	93197732	C	1.01 (0.97,1.05)	0.72	0.97 (0.89,1.06)	0.56	1.01 (0.97,1.05)	0.76
rs6465468	7	95169514	T	1.01 (0.97,1.05)	0.55	0.92 (0.84,1.01)	0.08	0.98 (0.94,1.02)	0.29
rs17405819	8	76806584	T	1.00 (0.96,1.04)	0.98	0.98 (0.90,1.08)	0.71	1.01 (0.97,1.06)	0.54
rs16907751	8	81375457	C	0.99 (0.93,1.06)	0.87	1.21 (1.04,1.42)	0.01	1.00 (0.94,1.07)	0.91
rs2033732	8	85079709	C	1.00 (0.95,1.04)	0.83	0.98 (0.89,1.08)	0.72	1.02 (0.98,1.07)	0.38
rs4740619	9	15634326	T	0.95 (0.91,0.98)	4.64E-03	1.01 (0.92,1.09)	0.90	1.04 (1.00,1.08)	0.07
rs10968576	9	28414339	G	1.00 (0.96,1.04)	0.93	0.99 (0.90,1.09)	0.92	1.01 (0.97,1.06)	0.51
rs6477694	9	111932342	C	1.01 (0.97,1.05)	0.72	0.97 (0.89,1.06)	0.49	1.00 (0.96,1.03)	0.85
rs1928295	9	120378483	T	1.02 (0.98,1.06)	0.31	0.95 (0.87,1.04)	0.25	1.05 (1.01,1.08)	0.01
rs10733682	9	129460914	A	0.97 (0.93,1.01)	0.12	0.99 (0.90,1.07)	0.75	0.99 (0.95,1.03)	0.63
rs7899106	10	87410904	G	0.99 (0.91,1.08)	0.77	1.32 (1.08,1.60)	5.29E-03	1.01 (0.93,1.09)	0.88
rs17094222	10	102395440	C	1.03 (0.98,1.08)	0.22	1.02 (0.90,1.14)	0.77	1.03 (0.98,1.08)	0.25
rs11191560	10	104869038	C	1.04 (0.97,1.12)	0.23	1.04 (0.89,1.22)	0.61	1.01 (0.96,1.07)	0.65
rs7903146	10	114758349	C	0.99 (0.95,1.03)	0.50	0.93 (0.84,1.03)	0.16	0.96 (0.92,1.00)	0.03
rs4256980	11	8673939	G	0.98 (0.94,1.02)	0.23	0.99 (0.90,1.08)	0.86	0.97 (0.93,1.01)	0.17
rs11030104	11	27684517	A	1.04 (0.99,1.09)	0.12	1.04 (0.93,1.16)	0.47	1.00 (0.96,1.04)	0.94

rs2176598	11	43864278	T	1.01 (0.96,1.05)	0.74	0.95 (0.86,1.05)	0.34	1.04 (0.99,1.09)	0.11
rs3817334	11	47650993	T	1.01 (0.98,1.05)	0.49	0.96 (0.88,1.04)	0.31	1.03 (0.99,1.06)	0.14
rs12286929	11	115022404	G	1.00 (0.96,1.04)	0.98	0.93 (0.85,1.01)	0.09	1.03 (0.99,1.07)	0.12
rs7138803	12	50247468	A	1.01 (0.98,1.05)	0.47	0.96 (0.88,1.05)	0.36	0.99 (0.95,1.03)	0.71
rs11057405	12	122781897	G	1.02 (0.96,1.08)	0.55	1.04 (0.90,1.20)	0.60	0.92 (0.86,0.97)	4.11E-03
rs9581854*	13	28017782	T	1.01 (0.96,1.06)	0.62	0.99 (0.89,1.09)	0.81	0.95 (0.91,1.00)	0.05
rs12429545	13	54102206	A	1.02 (0.96,1.07)	0.55	0.99 (0.87,1.13)	0.90	1.03 (0.97,1.09)	0.32
rs9540493	13	66205704	A	1.00 (0.96,1.04)	0.92	0.94 (0.86,1.02)	0.15	1.02 (0.98,1.06)	0.30
rs1441264	13	79580919	A	1.02 (0.98,1.06)	0.42	1.10 (1.01,1.21)	0.03	1.04 (1.00,1.08)	0.07
rs10132280	14	25928179	C	1.00 (0.96,1.04)	1.00	1.05 (0.95,1.15)	0.35	1.01 (0.96,1.05)	0.81
rs12885454	14	29736838	C	0.99 (0.95,1.03)	0.65	1.10 (1.00,1.21)	0.04	1.02 (0.98,1.06)	0.35
rs11847697	14	30515112	T	1.01 (0.93,1.11)	0.77	0.93 (0.73,1.20)	0.60	1.07 (0.97,1.17)	0.20
rs7141420	14	79899454	T	1.00 (0.96,1.03)	0.80	1.05 (0.96,1.14)	0.28	1.01 (0.97,1.05)	0.72
rs3736485	15	51748610	A	1.01 (0.97,1.05)	0.53	0.96 (0.88,1.05)	0.35	0.99 (0.95,1.03)	0.63
rs16951275	15	68077168	T	1.05 (1.01,1.10)	0.02	1.11 (0.99,1.23)	0.06	1.00 (0.96,1.04)	0.89
rs7164727	15	73093991	T	0.99 (0.96,1.03)	0.75	1.02 (0.92,1.12)	0.74	0.98 (0.95,1.02)	0.31
rs758747	16	3627358	T	1.03 (0.98,1.07)	0.23	1.05 (0.95,1.16)	0.30	1.05 (1.00,1.10)	0.06
rs12446632	16	19935389	G	1.05 (1.00,1.11)	0.07	0.98 (0.86,1.11)	0.71	0.97 (0.91,1.02)	0.22
rs2650492	16	28333411	A	1.03 (0.99,1.07)	0.21	1.01 (0.92,1.12)	0.78	1.05 (1.01,1.09)	8.75E-03
rs3888190	16	28889486	A	1.02 (0.98,1.06)	0.35	1.03 (0.95,1.13)	0.45	1.06 (1.03,1.10)	5.91E-04
rs4787491	16	30015337	G	1.01 (0.97,1.05)	0.64	0.95 (0.87,1.04)	0.27	1.03 (0.99,1.07)	0.12

rs9925964	16	31129895	A	1.05 (1.01,1.09)	9.26E-03	0.98 (0.90,1.07)	0.61	1.10 (1.06,1.14)	3.09E-07
rs2080454	16	49062590	C	1.00 (0.96,1.04)	0.98	1.03 (0.94,1.12)	0.57	0.99 (0.95,1.03)	0.60
rs1558902	16	53803574	A	1.01 (0.97,1.05)	0.60	1.04 (0.95,1.14)	0.34	1.06 (1.03,1.10)	3.94E-04
rs9914578	17	2005136	G	0.98 (0.94,1.03)	0.48	0.95 (0.85,1.05)	0.33	1.04 (0.99,1.10)	0.08
rs1000940	17	5283252	G	1.01 (0.97,1.05)	0.79	1.00 (0.90,1.09)	0.93	1.00 (0.95,1.04)	0.88
rs12940622	17	78615571	G	0.99 (0.96,1.03)	0.75	0.99 (0.90,1.07)	0.76	1.03 (0.99,1.07)	0.12
rs1808579	18	21104888	C	1.03 (1.00,1.07)	0.08	1.08 (0.99,1.17)	0.08	0.97 (0.94,1.01)	0.18
rs7239883	18	40147671	G	0.96 (0.92,1.00)	0.03	1.07 (0.98,1.16)	0.15	1.01 (0.97,1.05)	0.71
rs7243357	18	56883319	T	1.00 (0.95,1.05)	0.96	0.96 (0.85,1.08)	0.55	1.00 (0.95,1.05)	0.86
rs6567160	18	57829135	C	1.05 (1.01,1.10)	0.02	1.04 (0.94,1.15)	0.42	0.98 (0.94,1.02)	0.36
rs17724992	19	18454825	A	1.01 (0.96,1.05)	0.78	1.02 (0.92,1.13)	0.74	1.05 (1.00,1.10)	0.03
rs29941	19	34309532	G	1.01 (0.97,1.05)	0.53	0.91 (0.84,1.00)	0.05	0.99 (0.95,1.03)	0.55
rs2075650	19	45395619	A	1.04 (0.99,1.10)	0.15	0.96 (0.85,1.08)	0.51	0.99 (0.94,1.04)	0.67
rs2287019	19	46202172	C	1.02 (0.97,1.07)	0.36	1.01 (0.91,1.13)	0.84	0.96 (0.92,1.01)	0.13
rs3810291	19	47569003	A	1.03 (0.99,1.07)	0.17	0.97 (0.88,1.06)	0.47	0.94 (0.90,0.98)	6.69E-03
rs6091540	20	51087862	C	1.01 (0.97,1.05)	0.78	1.06 (0.96,1.16)	0.24	1.02 (0.98,1.06)	0.36
rs2836754	21	40291740	C	0.99 (0.95,1.03)	0.60	0.91 (0.84,1.00)	0.05	1.05 (1.02,1.09)	4.20E-03

Bp, base position; CHR, chromosome; CI, confidence interval; *proxy SNP for reported SNP (see S4 Table)

Table D. Proxy SNPs used to derive genetic instruments for BMI and psoriasis

Trait	Reported SNP	Proxy SNP	Measure of LD (r^2)
**BMI**	rs12016871	rs9581854	1.0
**Psoriasis**	rs887314	rs2510066	1.0
	rs41298997	rs17022427	1.0
	rs11531804	rs10816610	1.0
	rs144098432	rs565903	0.97
	rs142903734	rs79470265	1.0

BMI, body mass index; LD, linkage disequilibrium

Table E. Association of psoriasis genetic instruments with psoriasis in UK Biobank and HUNT data sets

Psoriasis variants reported by Tsoi et al (2017)

				Psoriasis (UK Biobank)		Psoriasis (HUNT)		Psoriasis (Tsoi 2017)	
**SNP**	**CHR**	**Position (bp)**	**Effect allele**	**OR (95% CI)**	***P* value**	**OR (95% CI)**	***P* value**	**OR (95% CI)**	***P* value**
rs7524364	1	8286009	A	1.08 (1.03,1.13)	1.20E-03	1.05 (0.94,1.17)	0.38	1.12 (1.07,1.17)	2.34E-07
rs10794648	1	24518206	C	1.08 (1.03,1.13)	8.00E-04	1.06 (0.96,1.17)	0.26	1.20 (1.15,1.25)	1.02E-17
rs6672420	1	25291010	T	1.10 (1.06,1.14)	7.00E-07	1.07 (0.98,1.17)	0.12	1.16 (1.12,1.20)	4.94E-17
rs113935720	1	67713346	T	1.26 (1.16,1.37)	2.80E-08	1.16 (0.94,1.43)	0.17	1.43 (1.33,1.54)	6.08E-21
rs10789285	1	69788482	G	1.02 (0.97,1.06)	0.43	1.00 (0.91,1.10)	0.96	1.07 (1.03,1.11)	8.35E-04
rs34517439	1	78450517	A	1.10 (1.04,1.17)	4.30E-04	1.09 (0.96,1.24)	0.2	1.19 (1.11,1.27)	1.19E-06
rs4845453	1	152591953	C	1.13 (1.09,1.18)	6.40E-10	1.12 (1.02,1.23)	0.02	1.20 (1.15,1.24)	3.70E-23
rs12118303	1	172675097	C	1.05 (1.01,1.11)	0.03	0.95 (0.85,1.07)	0.41	1.12 (1.07,1.17)	2.07E-07
rs3900909	1	197757846	G	1.09 (1.04,1.14)	1.40E-04	1.06 (0.95,1.18)	0.28	1.09 (1.05,1.14)	6.60E-05
rs17022427*	1	206654497	C	1.10 (1.05,1.15)	1.30E-05	NA	NA	1.11 (1.06,1.15)	5.91E-07
rs41298997	1	206655331	T	NA	NA	1.17 (1.06,1.29)	2.12E-03	1.13 (1.08,1.19)	4.01E-07
rs35194171	2	61072183	A	1.11 (1.07,1.15)	1.30E-07	1.17 (1.07,1.28)	4.13E-04	1.18 (1.14,1.22)	9.20E-21
rs4672505	2	62560332	A	1.07 (1.03,1.11)	3.50E-04	0.99 (0.91,1.09)	0.9	1.11 (1.08,1.15)	7.65E-10
rs17715343	2	163167746	C	1.24 (1.15,1.33)	2.90E-08	1.04 (0.88,1.23)	0.67	1.36 (1.27,1.45)	7.10E-20
rs13080782	3	16996623	G	1.08 (1.04,1.12)	3.50E-05	0.98 (0.90,1.07)	0.59	1.13 (1.09,1.17)	7.82E-12
rs1707602	3	101647309	G	1.07 (1.03,1.11)	1.40E-03	1.13 (1.03,1.25)	0.01	1.11 (1.07,1.15)	1.13E-08
rs28512356	3	189615475	C	1.09 (1.04,1.15)	2.30E-04	1.11 (0.99,1.24)	0.09	1.10 (1.04,1.15)	2.31E-04
rs112768831	5	40370724	C	1.06 (0.99,1.13)	0.1	1.21 (1.00,1.48)	0.06	1.12 (1.06,1.19)	2.43E-04
rs27044	5	96118852	G	1.09 (1.05,1.14)	1.80E-05	1.03 (0.93,1.13)	0.59	1.16 (1.12,1.20)	5.63E-16
rs1295685	5	131996445	G	1.15 (1.09,1.21)	1.10E-07	1.09 (0.98,1.22)	0.11	1.20 (1.15,1.25)	1.53E-16
rs74817271	5	150469973	A	1.41 (1.31,1.52)	2.10E-20	1.28 (1.06,1.53)	9.79E-03	1.62 (1.52,1.72)	2.73E-49
rs12188300	5	158829527	T	1.45 (1.37,1.53)	7.70E-40	1.50 (1.30,1.73)	2.78E-08	1.66 (1.57,1.76)	1.82E-69

rs9504361	6	577820	A	1.04 (1.00,1.08)	0.07	1.11 (1.02,1.21)	0.02	1.10 (1.06,1.14)	3.72E-08
rs7748720	6	20689945	A	1.09 (1.04,1.15)	1.30E-04	1.06 (0.95,1.17)	0.29	1.17 (1.12,1.22)	2.67E-14
rs9481169	6	111929862	T	1.24 (1.16,1.32)	1.20E-11	1.18 (1.01,1.38)	0.04	1.44 (1.36,1.52)	3.52E-39
rs582757	6	138197824	C	1.12 (1.07,1.16)	1.50E-07	1.09 (0.99,1.20)	0.07	1.20 (1.15,1.24)	6.55E-22
rs2451258	6	159506600	C	1.11 (1.07,1.15)	1.10E-07	1.07 (0.98,1.18)	0.12	1.10 (1.07,1.14)	2.70E-08
rs11767350	7	37385365	A	1.03 (0.99,1.06)	0.19	1.06 (0.97,1.16)	0.17	1.11 (1.07,1.15)	5.55E-09
rs11795343	9	32523737	T	1.13 (1.09,1.18)	2.30E-10	1.00 (0.91,1.09)	0.94	1.12 (1.08,1.16)	2.04E-10
rs10816610*	9	110781922	A	1.04 (1.00,1.08)	0.06	NA	NA	1.10 (1.06,1.13)	1.72E-07
rs11531804	9	110792282	T	NA	NA	1.03 (0.94,1.12)	0.53	1.11 (1.07,1.15)	1.43E-08
rs2944542	10	64369999	G	1.04 (1.00,1.08)	0.05	1.01 (0.92,1.10)	0.86	1.08 (1.04,1.12)	1.35E-05
rs2459446	10	75601596	C	1.09 (1.05,1.13)	5.00E-06	1.15 (1.05,1.25)	2.28E-03	1.12 (1.08,1.16)	1.06E-10
rs1108618	10	81043743	A	1.08 (1.04,1.12)	8.90E-05	1.09 (0.99,1.18)	0.07	1.12 (1.08,1.16)	3.56E-10
rs76959677	10	89824771	G	1.04 (0.95,1.13)	0.38	1.08 (0.85,1.39)	0.53	1.28 (1.16,1.41)	1.37E-06
rs61871342	10	102038641	G	1.05 (1.01,1.09)	0.02	1.05 (0.96,1.15)	0.29	1.10 (1.06,1.15)	2.63E-06
rs2510066*	11	64052447	C	1.05 (1.01,1.10)	7.30E-03	1.10 (1.01,1.21)	0.04	1.11 (1.07,1.15)	1.07E-08
rs4561177	11	109962432	A	1.03 (0.99,1.07)	0.12	1.04 (0.95,1.14)	0.35	1.14 (1.11,1.18)	5.97E-15
rs61907765	11	128391937	T	1.12 (1.07,1.17)	1.00E-06	1.19 (1.08,1.31)	7.39E-04	1.13 (1.09,1.18)	4.44E-10
rs11053802	12	10597207	T	1.08 (1.04,1.13)	8.50E-05	1.03 (0.94,1.13)	0.54	1.11 (1.06,1.16)	4.01E-06
rs57137641	12	56741228	G	1.24 (1.15,1.35)	1.60E-07	1.30 (1.08,1.56)	5.65E-03	1.42 (1.32,1.52)	1.42E-21
rs11065979	12	112059557	T	1.11 (1.07,1.15)	1.30E-07	1.02 (0.93,1.11)	0.71	1.08 (1.04,1.12)	1.38E-05
rs11059675	12	122668326	A	1.04 (1.01,1.08)	0.03	0.98 (0.90,1.07)	0.7	1.10 (1.06,1.14)	2.77E-06
rs73183592	13	40745693	G	1.04 (0.97,1.12)	0.26	1.05 (0.86,1.29)	0.63	1.22 (1.12,1.33)	4.59E-06
rs4942358	13	45321731	C	1.07 (1.03,1.12)	7.00E-04	1.06 (0.96,1.16)	0.25	1.10 (1.06,1.14)	5.22E-07
rs9513593	13	99950260	G	1.07 (1.02,1.12)	6.40E-03	1.04 (0.93,1.17)	0.5	1.12 (1.07,1.17)	2.75E-07
rs2145623	14	35839236	C	1.12 (1.08,1.17)	1.10E-08	1.08 (0.98,1.18)	0.11	1.16 (1.12,1.21)	4.11E-16
rs79470265*	14	98649129	C	0.94 (0.90,0.99)	0.02	NA	NA	0.89 (0.85,0.93)	5.87E-07

rs142903734	14	98668778	AAG	NA	NA	1.07 (0.96,1.2)	0.23	1.13 (1.08, 1.17)	7.08E-08
rs28624578	15	31637666	T	1.05 (1.00,1.11)	0.05	0.97 (0.86,1.08)	0.56	1.18 (1.11,1.24)	8.26E-09
rs413024	16	11354091	A	1.09 (1.04,1.13)	6.90E-05	1.05 (0.96,1.16)	0.28	1.13 (1.09,1.17)	4.85E-11
rs7184567	16	31021078	T	1.05 (1.02,1.10)	6.40E-03	0.95 (0.86,1.03)	0.22	1.14 (1.10,1.18)	1.49E-13
rs28998802	17	26124908	A	1.17 (1.11,1.23)	1.10E-09	1.12 (0.99,1.27)	0.07	1.24 (1.19,1.30)	3.91E-20
rs8070763	17	40536396	C	1.08 (1.04,1.12)	8.00E-05	1.06 (0.97,1.16)	0.2	1.11 (1.07,1.15)	3.01E-09
rs55823223	17	73890363	A	1.09 (1.04,1.15)	7.40E-04	0.99 (0.87,1.14)	0.9	1.14 (1.09,1.20)	8.39E-08
rs2304856	17	78175483	T	1.09 (1.05,1.13)	4.90E-06	1.16 (1.07,1.27)	7.14E-04	1.08 (1.04,1.12)	2.31E-04
rs559406	18	12857002	G	1.09 (1.05,1.13)	2.60E-06	1.09 (1.00,1.19)	0.04	1.10 (1.06,1.14)	5.72E-08
rs565903*	18	51777727	C	1.10 (1.06,1.14)	4.50E-06	NA	NA	1.10 (1.06,1.15)	7.34E-06
rs144098432	18	51816394	T	NA	NA	0.96 (0.87,1.06)	0.38	1.10 (1.07, 1.15)	9.88E-08
rs34536443	19	10463118	G	1.53 (1.37,1.71)	2.40E-14	1.55 (1.2,2.02)	1.02E-03	1.98 (1.78,2.21)	3.49E-35
rs4804528	19	10886206	G	1.05 (1.01,1.09)	6.70E-03	1.05 (0.96,1.15)	0.27	1.11 (1.07,1.15)	1.40E-09
rs492602	19	49206417	G	1.06 (1.02,1.10)	2.80E-03	1.05 (0.97,1.15)	0.23	1.11 (1.08,1.15)	4.24E-10
rs6067284	20	48574454	G	1.11 (1.07,1.16)	3.10E-08	1.08 (0.99,1.18)	0.07	1.17 (1.13,1.21)	3.44E-20
rs17812953	21	36488822	C	1.06 (1.00,1.11)	0.04	1.05 (0.93,1.19)	0.43	1.10 (1.05,1.16)	5.40E-05
rs5754387	22	21974703	C	1.10 (1.06,1.16)	1.90E-05	1.11 (1.01,1.22)	0.04	1.14 (1.09,1.19)	1.16E-09

Bp, base position; CHR, chromosome; CI, confidence interval; *proxy SNP for reported SNP (see Table D)

Table F. Association of psoriasis genetic instruments with BMI in UK Biobank and HUNT data sets

Psoriasis variants reported by Tsoi et al (2017)

				BMI (UK Biobank)		BMI (HUNT)	
**SNP**	**CHR**	**Position (bp)**	**Effect allele**	**Beta (95% CI)**	***P* value**	**Beta (95% CI)**	***P* value**
rs7524364	1	8286009	A	0.02 (0.00,0.05)	0.10	-0.07 (-0.18,0.04)	0.20
rs10794648	1	24518206	C	-0.03 (-0.05,0.00)	0.03	0.00 (-0.10,0.10)	0.98
rs6672420	1	25291010	T	0.02 (0.00,0.04)	0.04	0.08 (-0.01,0.17)	0.10
rs113935720	1	67713346	T	-0.01 (-0.05,0.03)	0.68	-0.25 (-0.46,-0.05)	0.01
rs10789285	1	69788482	G	0.02 (-0.01,0.04)	0.21	0.00 (-0.10,0.10)	1.00
rs34517439	1	78450517	A	0.18 (0.15,0.22)	2.78E-27	0.14 (0.00,0.28)	0.05
rs4845453	1	152591953	C	0.01 (-0.01,0.03)	0.29	0.04 (-0.05,0.14)	0.37
rs12118303	1	172675097	C	-0.01 (-0.04,0.02)	0.59	-0.06 (-0.17,0.06)	0.35
rs3900909	1	197757846	G	0.05 (0.03,0.08)	1.22E-04	0.08 (-0.03,0.19)	0.15
rs17022427*	1	206654497	C	0.03 (0.01,0.06)	7.93E-03	N/A	N/A
rs41298997	1	206655331	T	N/A	N/A	0.05 (-0.05,0.16)	0.31
rs35194171	2	61072183	A	0.01 (-0.01,0.03)	0.38	0.00 (-0.09,0.09)	0.98
rs4672505	2	62560332	A	0.00 (-0.02,0.03)	0.70	-0.04 (-0.14,0.05)	0.36
rs17715343	2	163167746	C	-0.02 (-0.06,0.02)	0.24	-0.07 (-0.25,0.10)	0.39
rs13080782	3	16996623	G	0.00 (-0.02,0.02)	0.95	0.03 (-0.06,0.12)	0.58
rs1707602	3	101647309	G	-0.01 (-0.03,0.01)	0.47	0.05 (-0.05,0.15)	0.29
rs28512356	3	189615475	C	0.02 (-0.01,0.04)	0.23	0.01 (-0.11,0.12)	0.91
rs112768831	5	40370724	C	-0.01 (-0.04,0.03)	0.77	0.14 (-0.05,0.33)	0.16
rs27044	5	96118852	G	-0.04 (-0.06,-0.01)	1.85E-03	0.08 (-0.02,0.18)	0.14
rs1295685	5	131996445	G	-0.04 (-0.07,-0.01)	2.91E-03	-0.06 (-0.17,0.05)	0.29
rs74817271	5	150469973	A	0.02 (-0.03,0.07)	0.36	0.03 (-0.18,0.24)	0.79
rs12188300	5	158829527	T	-0.05 (-0.09,-0.01)	7.35E-03	0.00 (-0.17,0.16)	0.95
rs9504361	6	577820	A	-0.02 (-0.04,0.00)	0.13	0.02 (-0.08,0.11)	0.74
rs7748720	6	20689945	A	-0.04 (-0.06,-0.01)	4.30E-03	-0.09 (-0.19,0.02)	0.11

rs9481169	6	111929862	T	-0.02 (-0.06,0.01)	0.20	-0.03 (-0.2,0.14)	0.73
rs582757	6	138197824	C	-0.01 (-0.03,0.01)	0.44	0.00 (-0.1,0.09)	0.94
rs2451258	6	159506600	C	0.01 (-0.02,0.03)	0.56	0.00 (-0.09,0.10)	0.94
rs11767350	7	37385365	A	0.02 (0.00,0.04)	0.06	-0.02 (-0.12,0.07)	0.60
rs11795343	9	32523737	T	0.03 (0.01,0.05)	5.19E-03	0.02 (-0.07,0.11)	0.64
rs10816610*	9	110781922	A	-0.01 (-0.03,0.01)	0.39	N/A	N/A
rs11531804	9	110792282	T	N/A	N/A	0.01 (-0.08,0.10)	0.87
rs2944542	10	64369999	G	-0.02 (-0.04,0.00)	0.06	-0.01 (-0.10,0.08)	0.82
rs2459446	10	75601596	C	0.03 (0.01,0.05)	0.01	0.07 (-0.02,0.16)	0.14
rs1108618	10	81043743	A	-0.05 (-0.07,-0.03)	3.56E-06	0.01 (-0.08,0.11)	0.75
rs76959677	10	89824771	G	0.00 (-0.05,0.05)	0.91	-0.07 (-0.34,0.19)	0.59
rs61871342	10	102038641	G	0.00 (-0.02,0.02)	0.82	0.07 (-0.02,0.16)	0.13
rs2510066*	11	64052447	C	-0.03 (-0.05,-0.01)	0.01	-0.04 (-0.13,0.05)	0.39
rs4561177	11	109962432	A	-0.01 (-0.03,0.01)	0.55	-0.09 (-0.18,0.01)	0.07
rs61907765	11	128391937	T	-0.01 (-0.03,0.02)	0.61	0.00 (-0.11,0.11)	0.99
rs11053802	12	10597207	T	0.01 (-0.02,0.03)	0.59	-0.04 (-0.13,0.06)	0.47
rs57137641	12	56741228	G	0.04 (0.00,0.08)	0.05	-0.10 (-0.27,0.07)	0.27
rs11065979	12	112059557	T	-0.05 (-0.07,-0.03)	1.44E-06	-0.05 (-0.14,0.05)	0.33
rs11059675	12	122668326	A	0.06 (0.04,0.08)	1.23E-07	0.03 (-0.06,0.12)	0.47
rs73183592	13	40745693	G	0.04 (0.00,0.08)	0.05	0.07 (-0.13,0.28)	0.47
rs4942358	13	45321731	C	-0.02 (-0.04,0.00)	0.10	-0.03 (-0.13,0.06)	0.52
rs9513593	13	99950260	G	0.01 (-0.01,0.04)	0.34	-0.12 (-0.24,0.00)	0.06
rs2145623	14	35839236	C	0.01 (-0.02,0.03)	0.49	-0.07 (-0.17,0.03)	0.16
rs79470265*	14	98649129	C	0.04 (0.01,0.07)	2.23E-03	N/A	N/A
rs142903734	14	98668778	AAG	N/A	N/A	0.06 (-0.05,0.18)	0.28
rs28624578	15	31637666	T	-0.03 (-0.05,0.00)	0.07	0.03 (-0.09,0.15)	0.61
rs413024	16	11354091	A	0.00 (-0.02,0.02)	0.90	-0.01 (-0.11,0.08)	0.77
rs7184567	16	31021078	T	0.07 (0.05,0.10)	4.12E-11	0.08 (-0.01,0.18)	0.08
rs28998802	17	26124908	A	0.00 (-0.03,0.03)	0.91	0.01 (-0.12,0.14)	0.86

rs8070763	17	40536396	C	0.00 (-0.02,0.02)	1.00	-0.06 (-0.16,0.04)	0.23
rs55823223	17	73890363	A	0.09 (0.06,0.12)	1.84E-08	0.20 (0.06,0.34)	4.54E-03
rs2304856	17	78175483	T	0.00 (-0.02,0.03)	0.73	0.00 (-0.09,0.09)	0.95
rs559406	18	12857002	G	0.00 (-0.03,0.02)	0.72	-0.12 (-0.21,-0.03)	8.93E-03
rs565903*	18	51777727	C	-0.01 (-0.04,0.01)	0.35	N/A	N/A
rs144098432	18	51816394	T	N/A	N/A	0.01 (-0.09,0.11)	0.84
rs34536443	19	10463118	G	-0.01 (-0.07,0.04)	0.59	-0.11 (-0.34,0.12)	0.36
rs4804528	19	10886206	G	-0.03 (-0.05,-0.01)	3.40E-03	0.01 (-0.08,0.10)	0.82
rs492602	19	49206417	G	-0.01 (-0.03,0.01)	0.26	0.05 (-0.04,0.14)	0.31
rs6067284	20	48574454	G	0.01 (-0.01,0.03)	0.44	0.01 (-0.08,0.10)	0.80
rs17812953	21	36488822	C	0.02 (-0.01,0.05)	0.11	-0.01 (-0.14,0.12)	0.87
rs5754387	22	21974703	C	0.01 (-0.02,0.04)	0.45	0.07 (-0.03,0.18)	0.16

Bp, base position; CHR, chromosome; CI, confidence interval; *proxy SNP for reported SNP (see Table D)

Table G. Reviewed studies where the association between psoriasis and BMI/obesity traits was tested using alternative models

Study	Age group	Association reported	Estimate
Duffy, 1993[1]	Adult	Mean difference in BMI (kg/m^2) (twin pairs) **MD [SEM]**	0.58 [0.38]
Naldi, 2005[2]	Adult	Odds of psoriasis in overweight participants **OR (95% CI)**	1.6 (1.1, 2.1)
Naldi, 2005[2]	Adult	Odds of psoriasis in obese participants **OR (95% CI)**	1.9 (1.2, 2.8)
Neimann (mild disease), 2006[3]	Adult	Odds of being overweight in psoriasis cases vs controls **OR (95% CI)**	1.12 (1.10, 1.14)
Neimann (mild disease), 2006[3]	Adult	Odds of obesity in psoriasis cases vs controls **OR (95% CI)**	1.27 (1.24, 1.31)
Niemann (severe disease), 2006[3]	Adult	Odds of being overweight in psoriasis cases vs controls **OR (95% CI)**	1.27 (1.14, 1.42)
Niemann (severe disease), 2005[3]	Adult	Odds of obesity in psoriasis cases vs controls **OR (95% CI)**	1.79 (1.55, 2.05)
Cohen, 2007[4]	Adult	Odds of obesity in psoriasis cases vs controls **OR (95% CI)**	1.30 (1.00, 1.70)

Setty, 2007[5]	Adult	Relative risk of psoriasis in overweight participants **RR (95% CI)**	1.40 (1.13, 1.73)
Setty, 2007[5]	Adult	Relative risk of psoriasis in obese participants **RR (95% CI)**	1.48 (1.15, 1.91)
Naldi, 2008[6]	Adult	Odds of psoriasis in overweight patients **OR (95% CI)**	1.6 (1.10, 2.20)
Naldi, 2008[6]	Adult	Odds of psoriasis in obese patients **OR (95% CI)**	1.70 (1.10, 2.60)
Boccardi, 2009[7]	Children	Odds of psoriasis in overweight individuals **OR (95% CI)**	2.55 (1.31, 4.96)
Driessen, 2009[8]	Adult	Mean BMI (kg/m^2) in psoriasis cases	28.5
Al Mutairi, 2010[9]	Adult	Odds of obesity in psoriasis cases vs controls **OR (95% CI)**	2.36 (1.93, 2.87)
Schmitt, 2010[10]	Adult	Odds of obesity in psoriasis cases vs controls **OR (95% CI)**	1.63 (1.39, 1.90)
Koebnick, 2011[11]	Children	Odds of psoriasis in overweight participants **OR (95% CI)**	1.31 (1.13, 1.49)

Koebnick, 2011[11]	Children	Odds of psoriasis in obese participants **OR (95% CI)**	1.39 (1.19, 1.63)
Zhu, 2012[12]	Children	Odds of being overweight in psoriasis cases vs controls **OR (95% CI)**	2.40 (1.2, 4.8)
Zhu, 2012[12]	Children	Odds of obesity in psoriasis cases vs controls **OR (95% CI)**	2.60 (1.00, 6.40)
Kumar, 2013[13]	Adult	Relative risk of psoriasis in overweight participants **RR (95% CI)**	1.21 (1.03, 1.43)
Kumar, 2013[13]	Adult	Relative risk of psoriasis in obese participants **RR (95% CI)**	1.63 (1.33, 2.00)
Tseng, 2013[14]	Adult	Odds of obesity in psoriasis cases vs controls **OR (95% CI)**	1.66 (1.17, 2.36)
Casagrande, 2014[15]	Adult	Odds of being overweight in psoriasis cases vs controls **OR (95% CI)**	2.03 (1.33, 3.12)
Casagrande, 2014[15]	Adult	Odds of obesity in psoriasis cases vs controls **OR (95% CI)**	1.59 (1.16, 2.17)
Harpsoe, 2014[16]	Adult	Hazard ratio for psoriasis in overweight individuals **HR (95% CI)**	1.38 (0.87, 2.20)

Harpsoe, 2014[16]	Adult	Hazard ratio for psoriasis in obese individuals **HR (95% CI)**	2.16 (1.25, 3.72)
Ma, 2014[17]	Adult	Mean weight (kg) of psoriasis cases and controls **Mean (SD)**	Cases: 64.5 [10.8] Controls: 56.8 [8.4]
Menegon, 2014[18]	Adult	Odds of being overweight in psoriasis cases vs controls **OR (95% CI)**	1.80 (1.10, 2.90)
Votrubova, 2014[19]	Adult	Odds of psoriasis with higher BMI **OR (95% CI)**	1.11 (1.04, 1.18)
Jacobi, 2015[20]	Adult	Mean BMI (kg/m^2) of psoriasis cases and controls	Cases: 28.0 Controls: 25.9
Diniz, 2016[21]	Adult	Median BMI (kg/m^2) of psoriasis cases and controls **Median (IQR)**	Cases: 26.7 (23.8, 30.7) Controls: 26.7 (24.5, 28.7)

CI, confidence interval; HR, Hazard ratio; IQR, interquartile range; MD, mean difference; OR, odds ratio; RR, relative risk; SD, standard deviation; SEM, standard error of the mean

1. Duffy DL, Spelman LS, Martin NG. Psoriasis in Australian twins. J Am Acad Dermatol. 1993;29: 428–434. doi:10.1016/0190-9622(93)70206-9
2. Naldi L, Chatenoud L, Linder D, Fortina AB, Peserico A, Virgili AR, et al. Cigarette smoking, body mass index, and stressful life events as risk factors for psoriasis: Results from an Italian case-control study. J Invest Dermatol. Elsevier; 2005;125: 61–67. doi:10.1111/j.0022-202X.2005.23681.x
3. Neimann AL, Shin DB, Wang X, Margolis DJ, Troxel AB, Gelfand JM. Prevalence of cardiovascular risk factors in patients with psoriasis. J Am Acad Dermatol. Mosby; 2006;55: 829–835. doi:10.1016/j.jaad.2006.08.040
4. Cohen A, Gilutz H, Henkin Y, Zahger D, Shapiro J, Bonneh D, et al. Psoriasis and the Metabolic Syndrome. Acta Derm Venereol. 2007;87: 506–509. doi:10.2340/00015555-0297
5. Setty AR, Curhan G, Choi HK. Obesity, waist circumference, weight change, and the risk of psoriasis in women: Nurses’ Health Study II. ArchInternMed. American Medical Association; 2007;167: 1670–1675. doi:10.1001/archinte.167.15.1670

6. Naldi L, Chatenoud L, Belloni A, Peserico A, Balato N, Virgili AR, et al. Medical history, drug exposure and the risk of psoriasis. Evidence from an Italian case-control study. Dermatology. Karger Publishers; 2008;216: 125-30–2. doi:10.1159/000111509
7. Boccardi D, Menni S, Vecchia C La, Nobile M, Decarli A, Volpi G, et al. Overweight and childhood psoriasis [Internet]. British Journal of Dermatology. Blackwell Publishing Ltd; 2009. pp. 484–486. doi:10.1111/j.1365-2133.2009.09276.x
8. Driessen RJB, Boezeman JB, Van De Kerkhof PCM, De Jong EMGJ. Cardiovascular risk factors in high-need psoriasis patients and its implications for biological therapies. J Dermatolog Treat. Taylor & Francis; 2009;20: 42–47. doi:10.1080/09546630802225702
9. Al-Mutairi N, Al-Farag S, Al-Mutairi A, Al-Shiltawy M. Comorbidities associated with psoriasis: An experience from the Middle East. J Dermatol. Blackwell Publishing Ltd; 2010;37: 146–155. doi:10.1111/j.1346-8138.2009.00777.x
10. Schmitt J, Ford DE. Psoriasis is independently associated with psychiatric morbidity and adverse cardiovascular risk factors, but not with cardiovascular events in a population-based sample. J Eur Acad Dermatology Venereol. Blackwell Publishing Ltd; 2010;24: 885–892. doi:10.1111/j.1468-3083.2009.03537.x
11. Koebnick C, Black MH, Smith N, Der-Sarkissian JK, Porter AH, Jacobsen SJ, et al. The Association of Psoriasis and Elevated Blood Lipids in Overweight and Obese Children. J Pediatr. Mosby; 2011;159: 577–583. doi:10.1016/j.jpeds.2011.03.006
12. Zhu KJ, He SM, Zhang C, Yang S, Zhang XJ. Relationship of the body mass index and childhood psoriasis in a Chinese Han population: a hospital-based study. J Dermatol. Blackwell Publishing Ltd; 2012;39: 181–183. doi:10.1111/j.1346-8138.2011.01281.x
13. Kumar S, Han J, Li T, Qureshi AA. Obesity, waist circumference, weight change and the risk of psoriasis in US women. J Eur Acad Dermatology Venereol. NIH Public Access; 2013;27: 1293–1298. doi:10.1111/jdv.12001
14. Tseng HW, Lin HS, Lam HC. Co-morbidities in psoriasis: A hospital-based case-control study. J Eur Acad Dermatology Venereol. Blackwell Publishing Ltd; 2013;27: 1417–1425. doi:10.1111/jdv.12028
15. Casagrande SS, Menke A, Cowie CC. No association between psoriasis and diabetes in the U.S. population. Diabetes Res Clin Pract. NIH Public Access; 2014;104: e58-60. doi:10.1016/j.diabres.2014.04.009
16. Harpsøe MC, Basit S, Andersson M, Nielsen NM, Frisch M, Wohlfahrt J, et al. Body mass index and risk of autoimmune diseases: A study within the Danish National Birth Cohort. Int J Epidemiol. Oxford University Press; 2014;43: 843–855. doi:10.1093/ije/dyu045
17. Ma L, Li M, Wang H, Li Y, Bai B. High prevalence of cardiovascular risk factors in patients with moderate or severe psoriasis in northern China. Arch Dermatol Res. Springer Berlin Heidelberg; 2014;306: 247–251. doi:10.1007/s00403-013-1437-3
18. Menegon DB, Pereira AG, Camerin AC, Cestari T. Psoriasis and comorbidities in a southern Brazilian population: A case-control study. Int J Dermatol. 2014;53: e518–e525. doi:10.1111/ijd.12186
19. Votrubova J, Juzlova K, Smerhovsky Z, Fialova J, Gopfertova D, Vojackova N, et al. Risk factors for comorbidities in Czech psoriatic patients: Results of a hospital-based case-control study. Biomed Pap. Biomedical Papers; 2014;158: 288–294. doi:10.5507/bp.2013.062
20. Jacobi A, Langenbruch A, Purwins S, Augustin M, Radtke MA. Prevalence of Obesity in Patients with Psoriasis: Results of the National Study PsoHealth3. Dermatology. Karger Publishers; 2015;231: 231–238. doi:10.1159/000433528
21. Diniz M dos S, Bavoso NC, Kakehasi AM, Lauria MW, Soares MMS, Machado-Pinto J, et al. Assessment of adiposity in psoriatic patients by dual energy X-ray absorptiometry compared to conventional methods. An Bras Dermatol. Sociedade Brasileira de Dermatologia; 2016;91: 150–155. doi:10.1590/abd1806-4841.20164082

Table H. One-sample MR estimates for the causal effect of higher BMI upon psoriasis within UK Biobank

Psoriasis measure	N (Cases / Controls)	OR (95% CI)	*P*-value
Any self-report	4,476 / 373,842	1.08 (1.03, 1.13)	0.002
Any HES defined	2,082 / 376,192	1.11 (1.04, 1.19)	0.002
Self-report only	3,537 / 372,655	1.07 (1.01, 1.13)	0.015
HES defined only	1,187 / 372,655	1.11 (1.02, 1.22)	0.021
Overlapping cases	882	1.11 (1.00, 1.24)	0.045

CI, confidence interval; HES, hospital episode statistics; OR, odds ratio.

Table I. Sensitivity analyses for the causal effect of BMI upon Psoriasis: OR per 1 unit increase in BMI (kg/m^2).

	Dataset	TSPS OR (95% CI)	IVW OR (95% CI)	MR-Egger OR (95% CI)	Weighted Median OR (95% CI)	Weighted MBE OR (95% CI)
Psoriasis	UK Biobank	1.08 (1.04, 1.13)	1.06 (1.02, 1.10)	1.04 (0.96, 1.13)	1.06 (1.00, 1.12)	1.06 (0.99, 1.14)
	HUNT	1.07 (0.98, 1.17)	1.09 (1.00, 1.19)	1.09 (0.94, 1.26)	1.12 (0.98, 1.27)	1.11 (0.96, 1.27)

CI, confidence interval; IVW, inverse variance weighted analysis; MBE, mode-based estimate; OR, odds ratio; TSPS, two-stage predictor substitution.

Table J. Sensitivity analyses for the causal effect of psoriasis genetic risk upon BMI

	Dataset	TSLS Estimate* (95% CI)	IVW Estimate** (95% CI)	MR-Egger Estimate** (95% CI)	Weighted Median Estimate** (95% CI)	Weighted MBE** (95% CI)
Psoriasis	UK Biobank	-0.59 (-4.68, 3.50)	0.00 (-0.05, 0.05)	-0.02 (-0.12, 0.08)	0.00 (-0.04, 0.04)	-0.01 (-0.05, 0.04)
	HUNT	0.65 (-2.10, 3.40)	0.03 (-0.05, 0.12)	0.03 (-0.09, 0.16)	0.03 (-0.09, 0.15)	0.04 (-0.08, 0.15)

* mean difference in BMI between psoriasis cases and controls
** change in BMI per doubling odds of psoriasis
CI, confidence interval; IVW, inverse variance weighted analysis; MBE, mode-based estimate; TSLS, two-staged least squares.

Table K. a) Association of psoriasis genetic instruments with BMI – estimates reported by Locke et al (2015)

Psoriasis variants reported by Tsoi et al (2017)

SNP	CHR	Position (bp)	Effect allele	Beta[a] (95% CI)	*P* value
rs10794648	1	24518206	T	-0.003 (-0.01,0.01)	0.58
rs6672420	1	25291010	A	-0.003 (-0.01,0.01)	0.46
rs10789285	1	69788482	T	0.003 (-0.01,0.01)	0.51
rs3900909	1	197757846	C	0.014 (0.00,0.02)	3.09E-03
rs4672505	2	62560332	G	-0.002 (-0.01,0.01)	0.63
rs17715343	2	163167746	G	0.004 (-0.01,0.02)	0.49
rs27044	5	96118852	C	-0.011 (-0.02,0.00)	7.14E-03
rs1295685	5	131996445	A	-0.002 (-0.01,0.01)	0.58
rs12188300	5	158829527	A	-0.006 (-0.02,0.01)	0.45
rs9504361	6	577820	G	-0.003 (-0.01,0.00)	0.43
rs7748720	6	20689945	G	-0.003 (-0.01,0.00)	0.47
rs582757	6	138197824	T	-0.002 (-0.01,0.01)	0.63
rs2451258	6	159506600	T	-0.002 (-0.01,0.01)	0.55
rs11795343	9	32523737	C	-0.001 (-0.01,0.01)	0.81
rs2944542	10	64369999	C	-0.001 (-0.01,0.01)	0.80
rs2459446	10	75601596	T	0.006 (0.00,0.01)	0.11
rs4561177	11	109962432	G	0.001 (-0.01,0.01)	0.81
rs11053802	12	10597207	C	-0.001 (-0.01,0.01)	0.88
rs4942358	13	45321731	A	-0.004 (-0.01,0.00)	0.29
rs9513593	13	99950260	A	0.003 (-0.01,0.01)	0.60
rs2145623	14	35839236	G	0.004 (0.00,0.01)	0.22

rs7184567	16	31021078	C	0.014 (0.01,0.02)	3.81E-04
rs2304856	17	78175483	C	0.000 (-0.01,0.01)	0.96
rs4804528	19	10886206	T	-0.005 (-0.01,0.00)	0.23
rs492602	19	49206417	A	-0.007 (-0.01,0.006)	0.03
rs17812953	21	36488822	T	0.003 (-0.01,0.01)	0.60
rs5754387	22	21974703	G	0.015 (0.01,0.02)	9.52E-04

[a]Estimate given for inverse-transformed BMI Bp, base position; CHR, chromosome; CI, confidence interval

Table K. b) Association of psoriasis genetic instruments (proxy variants) with BMI – estimates reported by Locke et al (2015)

Psoriasis variants reported by Tsoi et al (2017)

SNP	CHR	Position (bp)	Effect allele	Proxy SNP	Measure of LD (r^2)	Beta[a] (95% CI)	*P* value
rs7524364	1	8286009	G	rs12049468	0.82	-0.005 (-0.01,0.00)	0.32
rs113935720	1	67713346	C	rs11209026	1.00	0.006 (-0.01,0.02)	0.32
rs4845453	1	152591953	G	rs4845454	1.00	-0.002 (-0.01,0.01)	0.55
rs12118303	1	172675097	T	rs10798176	1.00	0.002 (-0.01,0.01)	0.71
rs17022427	1	206654497	T	rs12409804	1.00	0.000 (-0.01,0.01)	0.92
rs35194171	2	61072183	T	rs1177202	1.00	0.002 (-0.01,0.02)	0.70
rs13080782	3	16996623	A	rs3906814	1.00	0.001 (-0.01,0.01)	0.79
rs1707602	3	101647309	T	rs1707603	0.86	0.002 (-0.01,0.01)	0.53
rs28512356	3	189615475	A	rs1515490	0.83	-0.009 (-0.02,0.00)	0.05
rs112768831	5	40370724	A	rs12518245	1.00	-0.006 (-0.02,0.01)	0.36
rs74817271	5	150469973	G	rs17728338	1.00	-0.006 (-0.02,0.01)	0.38
rs9481169	6	111929862	G	rs13210247	0.85	-0.008 (-0.02,0.01)	0.32
rs11767350	7	37385365	G	rs17259252	0.98	0.007 (0.00,0.01)	0.08
rs10816610	9	110781922	C	rs10118193	1.00	0.007 (0.00,0.01)	0.07
rs1108618	10	81043743	G	rs1250546	0.87	0.011 (0.00,0.02)	7.47E-03
rs76959677	10	89824771	A	rs17346680	1.00	0.008 (-0.01,0.03)	0.47
rs61871342	10	102038641	A	rs17729876	0.98	0.000 (-0.01,0.01)	0.92
rs2510066	11	64052447	T	rs499425	0.96	-0.008 (-0.01,0.00)	0.05
rs61907765	11	128391937	C	rs11221332	0.94	-0.002 (-0.01,0.01)	0.62
rs57137641	12	56741228	A	rs11171803	1.00	-0.005 (-0.02,0.01)	0.39
rs11065979	12	112059557	C	rs11065987	0.80	0.015 (0.01,0.02)	1.18E-06
rs11059675	12	122668326	G	rs11059505	0.967	0.009 (0.00,0.02)	0.02
rs79470265	14	98649129	A	rs17701958	1.00	0.005 (0.00,0.01)	0.33
rs413024	16	11354091	G	rs243325	1.00	-0.001 (-0.01,0.01)	0.73
rs8070763	17	40536396	T	rs7217655	0.98	0.000 (-0.01,0.01)	0.92
rs55823223	17	73890363	G	rs3785437	0.81	-0.010 (-0.02,0.00)	0.09
rs565903	18	51777727	T	rs2849231	1.00	-0.004 (-0.01,0.00)	0.29
rs6067284	20	48574454	A	rs2235617	1.00	0.002 (-0.01,0.01)	0.56

[a]Estimate given for inverse-transformed BMI Bp, base position; CHR, chromosome; CI, confidence interval
