# Supplementary material for: Evidence of a causal relationship between body mass index and psoriasis: A mendelian randomization study
Source: PLoS Med. 2019 Jan 31;16(1):e1002739. doi: 10.1371/journal.pmed.1002739 (PMC6354959; doi:10.1371/journal.pmed.1002739)
Supplement: S1 STROBE Checklist — (DOC) [file pmed.1002739.s002.doc]

STROBE Statement—Checklist of items that should be included in reports of ***cohort studies***

|  | Item No | Recommendation |
| --- | --- | --- |
| **Title and abstract** | 1 | (*a*) Indicate the study’s design with a commonly used term in the title or the abstract **(Title and abstract)** |
|  |  | (*b*) Provide in the abstract an informative and balanced summary of what was done and what was found (“Methods and Findings” of the abstract) |
| Introduction | | |
| Background/rationale | 2 | Explain the scientific background and rationale for the investigation being reported **(2^nd^ and 3^rd^ paragraph of the Introduction)** |
| Objectives | 3 | State specific objectives, including any prespecified hypotheses **(final paragraph of the introduction)** |
| Methods | | |
| Study design | 4 | Present key elements of study design early in the paper (**methods section**) |
| Setting | 5 | Describe the setting, locations, and relevant dates, including periods of recruitment, exposure, follow-up, and data collection **(“Literature review and meta-analysis”, “study populations” and “clinical outcomes” section of the methods)** |
| Participants | 6 | (*a*) Give the eligibility criteria, and the sources and methods of selection of participants. Describe methods of follow-up (**“Study populations” and “clinical outcomes” section of the methods)** |
|  |  | (*b*) For matched studies, give matching criteria and number of exposed and unexposed **(N/A)** |
| Variables | 7 | Clearly define all outcomes, exposures, predictors, potential confounders, and effect modifiers. Give diagnostic criteria, if applicable (**“Study populations”, “clinical outcomes”, “confounder variables”, “observational analysis”, “Mendelian Randomization analysis” and “Fig 1” of the methods section)** |
| Data sources/ measurement | 8* | For each variable of interest, give sources of data and details of methods of assessment (measurement). Describe comparability of assessment methods if there is more than one group **(“Literature review and meta-analysis”, “study populations”, “clinical outcomes”, “genotyping”, “confounder variables”, “observational analysis”, “defining genetic instruments”)** |
| Bias | 9 | Describe any efforts to address potential sources of bias **(“Genotyping” and “sensitivity analysis” section of the methods)** |
| Study size | 10 | Explain how the study size was arrived at **(“Study populations” section of the methods)** |
| Quantitative variables | 11 | Explain how quantitative variables were handled in the analyses. If applicable, describe which groupings were chosen and why **(“Clinical outcomes” section of the methods)** |
| Statistical methods | 12 | (*a*) Describe all statistical methods, including those used to control for confounding **(“Observational analysis”, “Defining genetic instruments”, “Mendelian Randomization analysis”, “Sensitivity analysis”, “Reverse direction MR analysis” sections of the methods)** |
|  |  | (*b*) Describe any methods used to examine subgroups and interactions **(“Sensitivity analysis” section of the methods)** |
|  |  | (*c*) Explain how missing data were addressed **(“Genotyping” and “Defining genetic instruments” section of the methods)** |
|  |  | (*d*) If applicable, explain how loss to follow-up was addressed (N/A) |
|  |  | (*e*) Describe any sensitivity analyses **(“Sensitivity analysis” section of the methods)** |
| Results | | |
| Participants | 13* | (a) Report numbers of individuals at each stage of study—eg numbers potentially eligible, examined for eligibility, confirmed eligible, included in the study, completing follow-up, and analysed **(“Study populations”, “Defining genetic instruments” and table 1 of the methods section; “literature review”, fig 2, fig 3 and fig 4 of the results sections)** |
|  |  | (b) Give reasons for non-participation at each stage **(“Literature review and meta-analysis” section of the results)** |
|  |  | (c) Consider use of a flow diagram **(Table 1 of the methods section, fig 2, fig 3 and fig 4 of the results sections)** |
| Descriptive data | 14* | (a) Give characteristics of study participants (eg demographic, clinical, social) and information on exposures and potential confounders **(“Study populations” section of the methods)** |
|  |  | (b) Indicate number of participants with missing data for each variable of interest **(Table 1 of the methods section** |
|  |  | (c) Summarise follow-up time (eg, average and total amount) **(N/A)** |
| Outcome data | 15* | Report numbers of outcome events or summary measures over time **(Results section)** |
| Main results | 16 | (*a*) Give unadjusted estimates and, if applicable, confounder-adjusted estimates and their precision (eg, 95% confidence interval). Make clear which confounders were adjusted for and why they were included **(Results section)** |
|  |  | (*b*) Report category boundaries when continuous variables were categorized **(“Observational analysis” section of the results)** |
|  |  | (*c*) If relevant, consider translating estimates of relative risk into absolute risk for a meaningful time period **(N/A)** |
| Other analyses | 17 | Report other analyses done—eg analyses of subgroups and interactions, and sensitivity analyses **(“Observational analysis”, “Mendelian Randomization” and “Reverse MR analysis – genetic liability for psoriasis upon BMI” sections of the results)** |
| Discussion | | |
| Key results | 18 | Summarise key results with reference to study objectives **(Discussion)** |
| Limitations | 19 | Discuss limitations of the study, taking into account sources of potential bias or imprecision. Discuss both direction and magnitude of any potential bias **(Discussion)** |
| Interpretation | 20 | Give a cautious overall interpretation of results considering objectives, limitations, multiplicity of analyses, results from similar studies, and other relevant evidence **(Discussion)** |
| Generalisability | 21 | Discuss the generalisability (external validity) of the study results **(Discussion** |
| Other information | | |
| Funding | 22 | Give the source of funding and the role of the funders for the present study and, if applicable, for the original study on which the present article is based **(Acknowledgements)** |

*Give information separately for exposed and unexposed groups.

**Note:** An Explanation and Elaboration article discusses each checklist item and gives methodological background and published examples of transparent reporting. The STROBE checklist is best used in conjunction with this article (freely available on the Web sites of PLoS Medicine at http://www.plosmedicine.org/, Annals of Internal Medicine at http://www.annals.org/, and Epidemiology at http://www.epidem.com/). Information on the STROBE Initiative is available at http://www.strobe-statement.org.
